# Heat-Induced Release of Epigenetic Silencing Reveals the Concealed Role of an Imprinted Plant Gene

**DOI:** 10.1371/journal.pgen.1004806

**Published:** 2014-11-20

**Authors:** Diego H. Sanchez, Jerzy Paszkowski

**Affiliations:** University of Geneva, Laboratory of Plant Genetics-Sciences III, Genève, Switzerland; Whitehead Institute for Biomedical Research, United States of America

## Abstract

Epigenetic mechanisms suppress the transcription of transposons and DNA repeats; however, this suppression can be transiently released under prolonged heat stress. Here we show that the *Arabidopsis thaliana* imprinted gene *SDC*, which is silent during vegetative growth due to DNA methylation, is activated by heat and contributes to recovery from stress. *SDC* activation seems to involve epigenetic mechanisms but not canonical heat-shock perception and signaling. The heat-mediated transcriptional induction of *SDC* occurs particularly in young developing leaves and is proportional to the level of stress. However, this occurs only above a certain window of absolute temperatures and, thus, resembles a thermal-sensing mechanism. In addition, the re-silencing kinetics during recovery can be entrained by repeated heat stress cycles, suggesting that epigenetic regulation in plants may conserve memory of stress experience. We further demonstrate that *SDC* contributes to the recovery of plant biomass after stress. We propose that transcriptional gene silencing, known to be involved in gene imprinting, is also co-opted in the specific tuning of *SDC* expression upon heat stress and subsequent recovery. It is therefore possible that dynamic properties of the epigenetic landscape associated with silenced or imprinted genes may contribute to regulation of their expression in response to environmental challenges.

## Author Summary

In plants, expression of certain imprinted genes is restricted to embryo nourishing tissue, the endosperm. Since these genes are silenced by epigenetic mechanisms during vegetative growth, it has been assumed that they have no role in this phase of the plant life cycle. Here, we report on heat-mediated release of epigenetic silencing and ectopic activation of the *Arabidopsis thaliana* endosperm-imprinted gene *SDC*. The stress induced activation of *SDC* involves epigenetic regulation but not the canonical heat-shock perception and signaling, and it seems to be required for efficient growth recovery after the stress. Our results exemplify a potential concealed role of an imprinted gene in plant responses to environmental challenges.

## Introduction

It has been long recognized that transcriptional gene silencing (TGS) in plants is associated mainly with increased levels of DNA methylation [1], [2]. DNA methylation is found in cytosines (C) residing in CG, CHG and CHH sequence contexts (where H stands for A, T or C). *Methyltransferase 1* (MET1) perpetuates CG methylation patterns during DNA replication. Cytosine methylation in CHG and CHH sequences is mediated by *Chromomethylase 3* (CMT3) and *Chromomethylase 2* (CMT2), respectively [3]–[6]. Cytosine methylation in asymmetric CHH sequences cannot be maintained in a replicative manner and the RNA-dependent DNA methylation (RdDM) pathway leads to their methylation *de novo* through sequence-specific targeting with small interfering RNAs, and thus the mitotic persistence of TGS [7]. *De novo* DNA methylation occurs in all sequence contexts and is mainly catalyzed by *Domains Rearranged Methyltransferase 2* (DRM2) [3], [8].

TGS is involved in the epigenetic suppression of invading DNA, such as that of pathogens but also of endogenous transposons, which threaten genome stability by their mutational capacity and deleterious regulatory effects on neighboring genes [9], [10]. However, TGS is also involved in genomic imprinting, i.e. allele-specific expression dependent on the parent-of-origin. The expression of some imprinted genes in plants is restricted to seed endosperm and is associated with silencing during somatic growth [11]. Such strict developmental regulation of imprinted gene expression is critical for seed and plant development. In *Arabidopsis thaliana*, aberrant expression of imprinted genes such as *Medea* (*MEA*) and *Fertilization Independent Seed 2* (*FIS2*) has strong phenotypic consequences that lead to seed abortion [12]. Ectopic expression of imprinted genes during vegetative growth may also have phenotypic consequences. For example, the imprinted *A. thaliana* gene *SDC* is epigenetically silenced in somatic tissues due to DNA methylation targeted by the RdDM pathway to tandem-repeats within its promoter. This locus is highly activated in particular combinations of TGS mutants such as *drm1/drm2/cmt3* and *ddm1/drd1*, which results in leaf curling and plant dwarfism [5], [13].

Although epigenetic mechanisms can suppress transcription at ambient temperatures, it was reported recently that transcriptional activation can occur transiently during prolonged exposure to heat [14]–[17]. The degree of activation was proportional to the duration of the stress and was associated with decreased nucleosome occupancy and resulting chromatin decondensation. Importantly, chromatin assembly factors restored silencing within 48 h after heat stress [14]. Here, we report on heat stress-mediated ectopic activation of the imprinted *SDC* gene in vegetative tissues. The stress-triggered transcriptional response of *SDC* occurred particularly in young developing leaves and the kinetics of re-silencing could be entrained by repeated heat stress cycles. We provide evidence for a physiological role of this unexpected regulation of an imprinted gene during recovery from heat stress.

## Results

**Figure 1:**
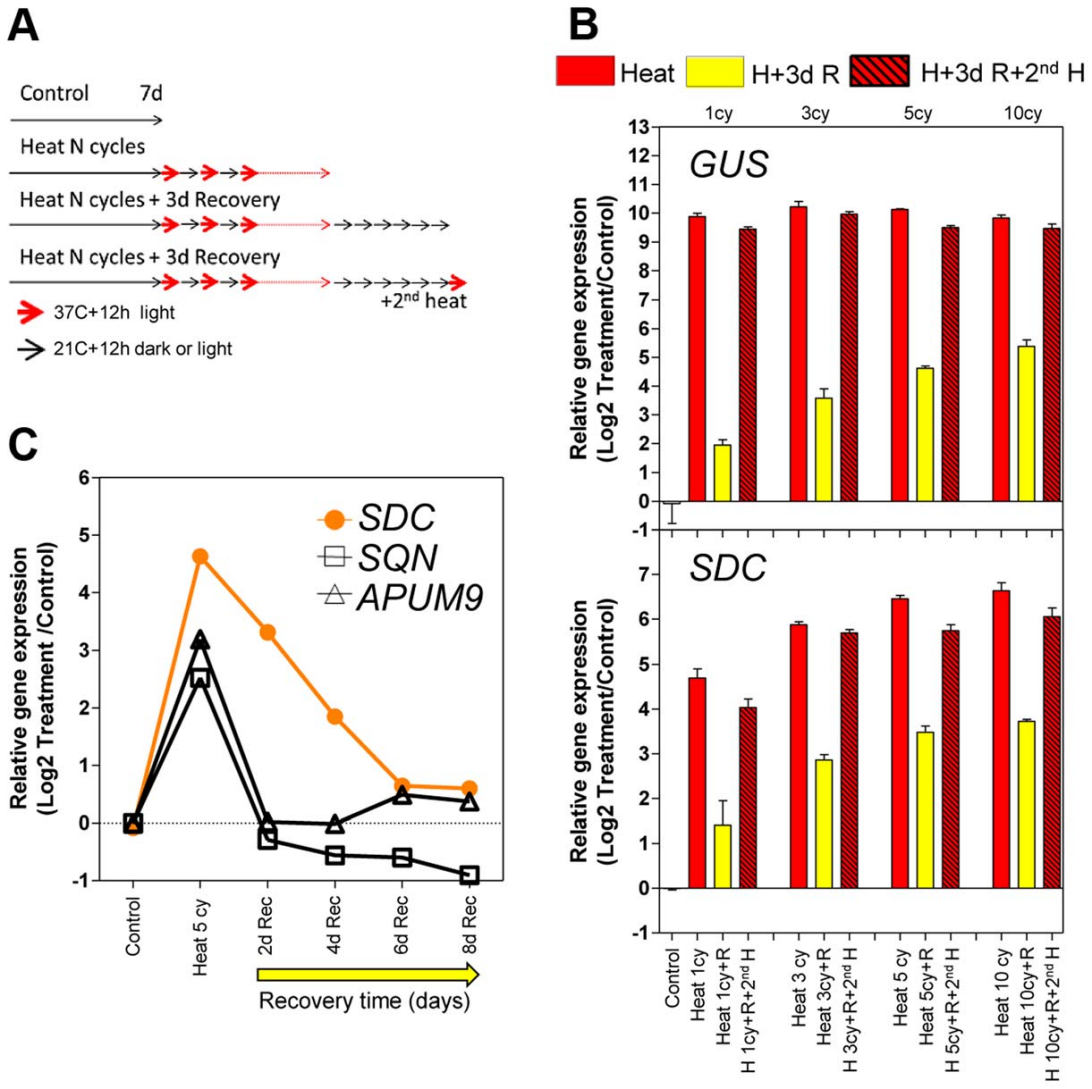
Transcriptional memory of the heat-induced *SDC* activation state. **A**, Design of the heat-entrainment experiment. Seven-day-old *A. thaliana* seedlings were subjected to standard conditions, heat cycle entrainment (day 37°C and night 21°C, 12/12 h), heat cycle entrainment +3 days recovery at 21°C, and heat cycle entrainment +3 days recovery +2^nd^ heat treatment (day 37°C,12 h). Heat entrainment was performed for 1, 3, 5 or 10 cycles. **B**, Transcript levels of *L5-GUS* and *SDC* loci during recovery from heat-induced release of silencing. Bars represent means ± SE as a log2 ratio with the non-treated control condition (i.e., control  = 0); replicated samples were pooled from 40–60 whole seedlings. **C**, Kinetics of transcript level re-silencing of the partially silent genes during the recovery phase following a 5 heat-cycle entrainment. Dots represent mean gene expression as a log2 ratio with the non-treated control condition (i.e., control  = 0); replicated samples were pooled from 40–60 whole seedlings.

To analyze the heat-mediated release of TGS, we used a transgenic line carrying a silent *35S::GUS* construct, referred to as *L5-GUS* [2]. The transcription of this transgene is repressed by DNA methylation of the promoter, and its silencing is released in several epigenetic mutants such as *mom1, ddm1 and met1* [2], [14]. *L5-GUS A. thaliana* seedlings were subjected to an acclimation treatment consisting of a varying number of diurnal heat cycles as entrainment. Each cycle comprised 12 h at 37°C in the light and 12 h at 21°C in the dark. This experimental design with elevated temperature associated with light periods closely models natural growth conditions, when plants experience high temperatures mostly during the day. The entrainment was followed by a recovery period of 3 days at 21°C with a 12 h/12 h light/dark cycle. After recovery, an additional heat cycle (second stress) was applied to a subset of the entrained seedlings (Figure 1A). As expected, *GUS* transcription was released upon heat stress but resulted in similar transcript levels between each heat cycle and the second stress. However, *GUS* mRNA levels during recovery showed a stepwise increase proportional to the number of heat stress cycles (Figure 1B). This result suggests either transcriptional memory related to the previous heat-induced release of silencing or merely the physiological consequence of a higher perceived stress dose. To distinguish between these possibilities, we examined the transcriptional regulation of typical heat stress-responsive genes after the entrainment. These loci did not show an *L5-GUS-*related pattern of mRNA accumulation, during either activation or recovery (Figure S1), indicating that the transcriptional consequences of entrainment were specific to the epigenetically regulated *L5-GUS* transgene.

In a search for protein-coding genes displaying responses similar to the *L5-GUS* transgene, we examined a subset of loci known to be transcriptionally suppressed by epigenetic modification of their promoters but activated in epigenetic mutants or under heat stress [16], [18]–[20]. We tested the heat stress entrainment of the genes *SDC* (AT2G17690), *SQN* (AT2G15790), and *APUM9* (AT1G35730). Of these three candidates, only *SDC* showed a response pattern similar to *L5-GUS* (Figure 1B and S1). We hypothesized that for both *L5-GUS* and *SDC*, the positive correlation between elevated transcript levels during recovery and the number of heat stress cycles reflects an altered speed of re-silencing as a consequence of the entrainment (Figure S2). To test this possibility, we focused on *SDC* re-silencing kinetics. Indeed, after entrainment by 5 heat cycles, *SDC* transcripts displayed significantly slower re-silencing kinetics than *SQN* and *APUM9* (Figure 1C). In this experiment, we also assayed some heat-induced transposable elements from different families. We observed cases of fast and slow re-silencing, suggesting that both patterns are possible in TGS targets (Figure S2).

**Figure 2:**
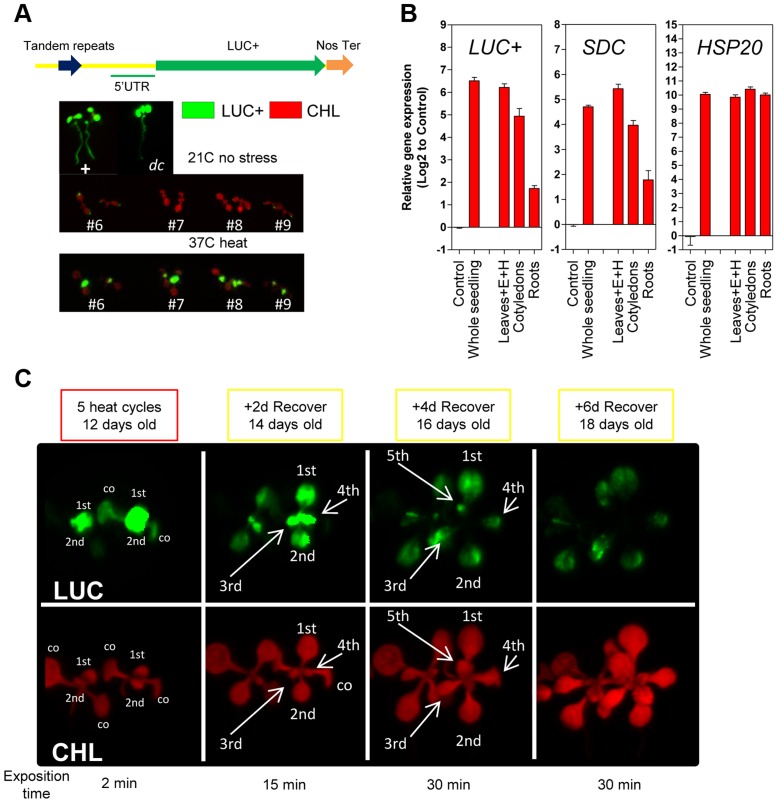
Tissue-specific heat-induced release of *SDC* gene silencing. **A**, *In vivo* luciferase activity in transgenic seedlings. Top: Positive control *UBQ3::LUC+* and transgenic *dc* mutant (*-1200SDCProm::LUC+*). Bottom: #6, #7, #8 and #9 represent independent Col-0 transgenic lines (*-1200SDCProm::LUC+*) under standard conditions or heat stress. **B**, Transcript levels of *LUC+*, *SDC* and *HSP20* in different tissues of transgenic Col-0 seedlings (*-1200SDCProm::LUC+*) after a 5 heat cycle entrainment. Bars represent means ± SE as a log2 ratio with the non-treated control condition (i.e., control  = 0); replicated samples were pooled from 20–30 seedlings (E: epicotyl, H: hypocotyl). **C**, *In vivo* luciferase activity in 7-days-old Col-0 transgenic seedlings (-*1200SDCProm::LUC+*) after an entrainment of 5 heat-cycles and varying recovery times. LUC: luciferase signal, CHL: chlorophyll signal. Arrows depict specific leaves; co: cotyledon, and 1st, 2nd, 3th, 4th, 5th represent developing true leaves.

The *SDC* promoter contains tandem-repeats targeted by the TGS machinery and it is possible that this particular promoter structure contributes to the observed transcriptional entrainment. To address this, we constructed a vector containing the *SDC* promoter linked to the luciferase reporter (*-1200PromSDC::LUC+*) and transformed *A. thaliana* Col-0 wild type and the *drm2-2/cmt3-11* double mutant (referred to as *dc*), which is deficient in RdDM and CMT3-mediated DNA methylation responsible for *SDC* silencing [13]. In *dc* transgenic plants under control conditions, a strong luciferase signal was recorded from *-1200PromSDC::LUC+* throughout entire seedlings, implying that the *SDC* promoter does not require heat for activation (Figure 2A). In the Col-0 transgenic plants, *-1200PromSDC::LUC+* was transcriptionally suppressed but remained responsive to activation by heat stress (Figure 2A), demonstrating that DNA methyltransferases targeted the *-1200PromSDC::LUC+* transgene and the promoter of the endogenous *SDC* gene in a similar way. Closer examination of the luciferase signals showed them to be highest in young true leaves, lower in cotyledons, and absent from roots (Figure 2A). To determine whether the transcriptional regulation of the *-1200PromSDC::LUC+* transgene indeed reflects the heat-induced activation and developmental regulation of the *SDC* gene, we compared their relative transcript levels in various tissues of seedlings subjected to heat stress. The levels and tissue distribution of mRNA were very similar for both transgenic and endogenous loci, with highest heat induction in young leaves and the lowest in roots. Moreover, they clearly differed from the expression patterns of typical heat-responsive genes, which are induced ubiquitously throughout all seedlings tissues (Figure 2B and S3A). After entrainment for 5 heat-cycles, the 1^st^ and 2^nd^ leaves of *-1200PromSDC::LUC+* Col-0 plants showed high transgenic transcript levels and high luciferase signals, decreasing during the recovery phase with kinetics similar to that observed previously for *SDC* (Figure S3B). Interestingly, leaves 3 to 5 developed during the 4 days of recovery and also showed luciferase signals (Figure 2C). Furthermore, when older plants were subjected to heat stress, marked luciferase signals were found mostly in developing leaves 5 and 6 but were largely absent from older leaves (1 to 4) developed before stress application (Figure S3C). This indicated that not fully expanded young leaves, or possibly even their primordia in the apical meristem, respond predominantly to heat stress by activation of the *SDC* promoter. Moreover, the slow re-silencing kinetics of endogenous *SDC* and the luciferase signals from -*1200PromSDC::LUC+* Col-0 plants suggest that the acquired active state is maintained during the maturation of leaves when recovering from stress.

We determined that the number of tandem repeats within the *SDC* promoter in a subset of *A. thaliana* accessions is typically seven or eight. Therefore, we compared the kinetics of heat-induced *SDC* activation and re-silencing in these two categories by applying 5 heat cycles and 3 days of recovery. Across all accessions tested, the *SDC* gene was silent under control conditions and activated by heat stress, suggesting an evolutionary conservation of the heat-induced transcriptional response (Figure S4). However, the relative transcript levels induced by heat stress and their persistence during recovery varied significantly between accessions (Figure S4). The observed differences in *SDC* regulation could not be attributed to the different number of repeats, demonstrating that a slight variation in the genetic constitution of the promoter does not determine differences in the kinetics of heat-induced release of *SDC* silencing.

**Figure 3:**
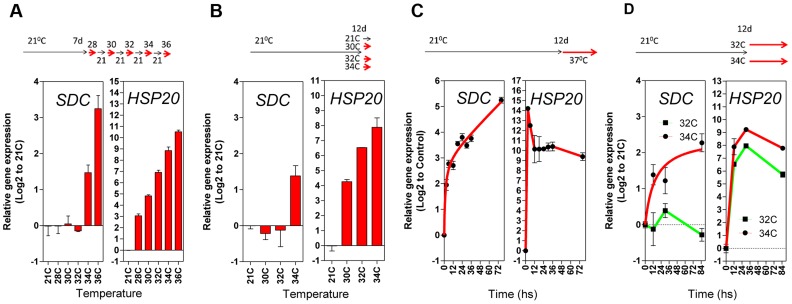
Transcriptional patterns of *SDC* expression under heat compared to a typical heat-shock gene. **A** and **B**, Thermal threshold of transcriptional activation for *SDC* and *HSP20*. **A**, Seedlings were grown under standard conditions for 7 days and then subjected to daily increases in temperature. **B**, Seedlings were grown under standard conditions for 12 days and then subjected to a one-step increase in temperature for 12 h. **C** and **D**, **H**eat time-course for transcript levels of *SDC* and *HSP20*. **C**, Seedlings were grown under standard condition for 7 days and then subjected to constant heat for at least 74 h. **D**, Seedlings were grown under standard condition for 12 days and then subjected to a one-step increase in temperature to a constant 32°C or 34°C. Bars and dots represent the means ± SE as a log2 ratio with the non-treated control conditions (i.e., control  = 0); replicated samples were pooled from 40–60 seedlings. In all cases, the experimental design is shown at the top.

Next, we compared the pattern of transcriptional responses of *SDC* to typical heat-responsive loci. The thermal threshold of their activation was tested with daily increases in the ambient temperature of growing seedlings (between 28°C and 36°C, Figure 3A). Heat-responsive genes were already activated transcriptionally when plants were moved from 21°C to 28°C, and transcript levels further increased stepwise with increasing temperature (Figure 3A and Figure S5A). However, the *SDC* locus displayed a distinct thermal threshold for activation within a window of 2°C, from 32°C to 34°C (Figure 3A). A further experiment using a single-step change in temperature yielded similar results, demonstrating that the narrow thermal threshold was independent of the temperature applied on the previous day (Figure 3B and Figure S5B). In a heat time-course (76 h at a constant 37°C), expression of the typical heat-responsive genes peaked rapidly 3 h after the start of the treatment and remained at high levels relative to the control conditions. However, the accumulation of *SDC* transcripts showed no peak but developed in proportion to the length of the heat stress (Figure 3C and S5C), similar to the responses of other epigenetically regulated loci [14], [16], [17]. Notably, *SDC* activation occurred only when the heat stress reached the thermal threshold, unlike the heat-responsive genes (Figure 3D and Figure S5D). Overall, similar activation patterns to *SDC* were observed for the luciferase transcript from -*1200PromSDC::LUC+* Col-0 transgene (Figure S5A and C). Taken together, these data support the notion that the particular transcriptional regulation of *SDC* takes place independently of canonical heat-shock perception and signaling.

**Figure 4:**
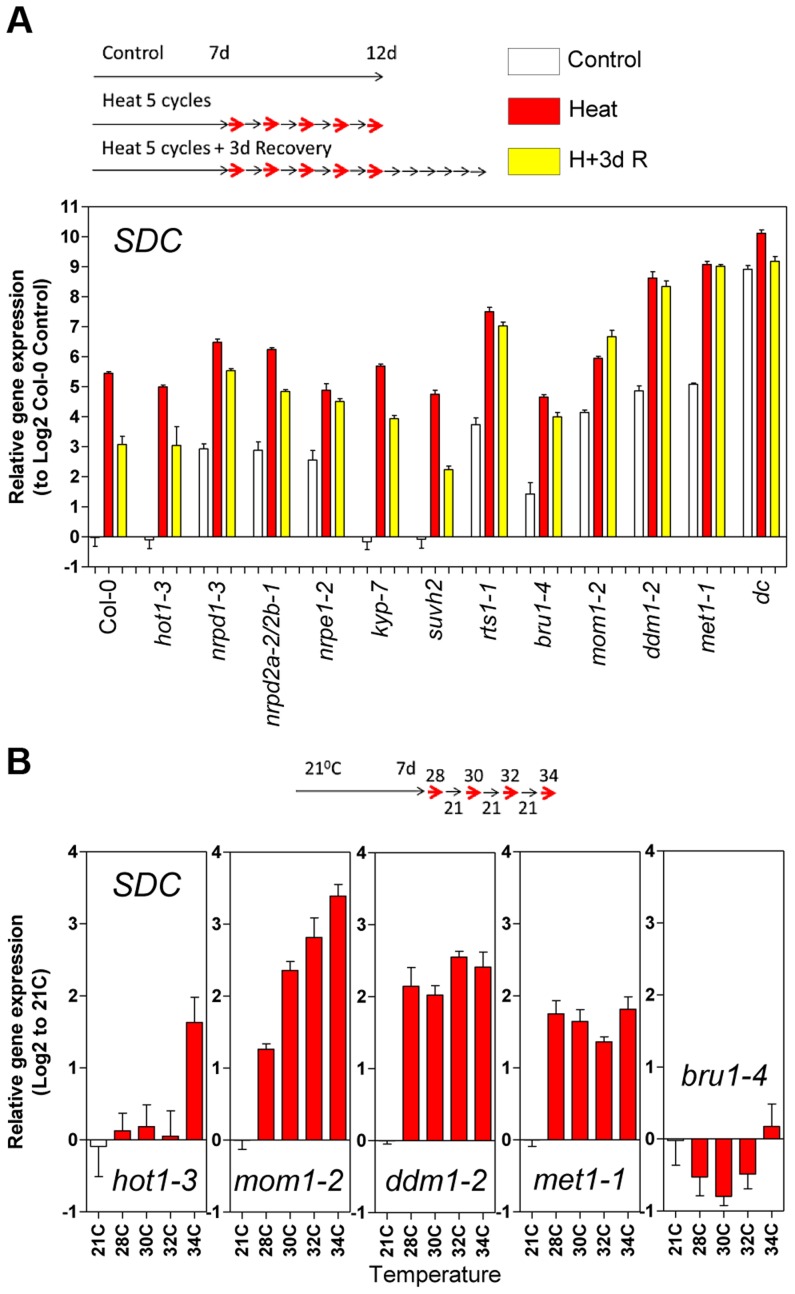
Epigenetic regulation of heat-induced *SDC* release from silencing. **A**, Relative levels of *SDC* transcripts in *A. thaliana* mutants under control conditions, after a 5 heat cycle entrainment and a 5 heat cycle entrainment +3 days recovery. Bars represent means ± SE as a log2 ratio with the non-treated wild-type Col-0 control conditions (i.e., Col-0 control  = 0); replicated samples were pooled from 40–60 whole seedlings. **B**, Thermal threshold of transcriptional activation of *SDC* in epigenetic mutants. Bars represent means ± SE as a log2 ratio with the non-treated control conditions (i.e., control  = 0); replicated samples were pooled from 40–60 seedlings.

To assess whether compromised heat stress tolerance contributes to *SDC* regulation, we tested the heat stress hypersensitive mutant *hot1-3*, which is impaired in the *Heat Shock Protein 101* (*HSP101*) [21]. Heat-induced *SDC* transcription, re-silencing and thermal-threshold patterns in *hot1-3* were identical to that in wild-type plants (Figure 4A and B). Moreover, experiments performed with this mutant should be indicative of the relative level of heat stress perceived by plants under our experimental conditions. Transcriptional regulation of typical heat-responsive genes was not altered in *hot1-3*, consistent with the heat stress levels applied in our experimental conditions being relatively low (Figure S6).

Since transcription of *SDC* is suppressed during vegetative growth by DNA methylation and possibly other epigenetic mechanisms, we examined *SDC* transcriptional heat-stress responses, re-silencing kinetics, and thermal threshold properties in mutants impaired in various aspects of TGS. The transcriptional heat-stress responses of *SDC* observed in these epigenetic mutants could be divided into three categories: a) not different to the wild type (*kyp-7* and *suvh2*); b) almost complete release of *SDC* silencing and thus loss of additional transcriptional activation induced by heat (*dc*); c) partial release of *SDC* silencing under control conditions but heat-stress induction maintained (*nrpd1-3*, *nrpd2a-2/2b-1*, *nrpe1-2*, *rts1-1*, *bru1-4*, *mom1-2*, *ddm1-2* and *met1-1*) (Figure 4A). In the latter category, re-silencing of *SDC* to control levels was largely impaired in some mutants, especially in *mom1-2*, *ddm1-2* and *met1-1* (Figure 4A). Importantly, typical heat-responsive genes displayed unaltered transcriptional responses across all the mutants tested (Figure S6), indicating that canonical heat-shock signaling is not influenced by the epigenetic mechanisms examined here. We further tested whether the specific thermal threshold for *SDC* activation is affected by the mutations in epigenetic regulation. The threshold was clearly disturbed in these mutants and was moved towards lower (*mom1-2*, *ddm1-2* and *met1-1*) or higher temperatures (*bru1-4*) (Figure 4B). Because *MOM1* and *BRU1* influence the stability of particular chromatin states at target loci [18], [20], [22] but their mutated alleles do not affect DNA methylation within the *SDC* promoter [23], their effect on the thermal threshold is consistent with the chromatin state *per se* being a candidate for the threshold regulation. We examined DNA methylation and a set of histone modifications within the *SDC* locus in control and heat-stressed plants but found no evidence for major stress-induced alterations in these epigenetic marks (Figure S7 and S8). Therefore other as yet undefined chromatin properties may determine the narrow temperature range of the transcriptional activation of the *SDC* gene.

*SDC* is an imprinted locus, with maternal allele activation during endosperm development and otherwise silent during the entire vegetative growth [24], [25]. To test whether additional endosperm-imprinted genes may be subjected to stress-triggered transcriptional activation, we examined 93 genes for *SDC*-like transcriptional activation triggered by environmental stress. These 93, selected from 114 previously confirmed endosperm-imprinted genes [24], were represented on the Affymetrix GeneChip ATH1 used in experiments with plants subjected to various stress conditions [26]. A number of stresses including cold, osmotic stress, salinity, wounding, oxidative stress, UV-B irradiation and heat were able to induce transcription of many of these genes during vegetative growth (Figure S9). Although these results suggest that they may contribute to the stress responses and possibly stress tolerance, this hypothesis requires further experimental support, comparable to the study on the *SDC* gene described below.

**Figure 5:**
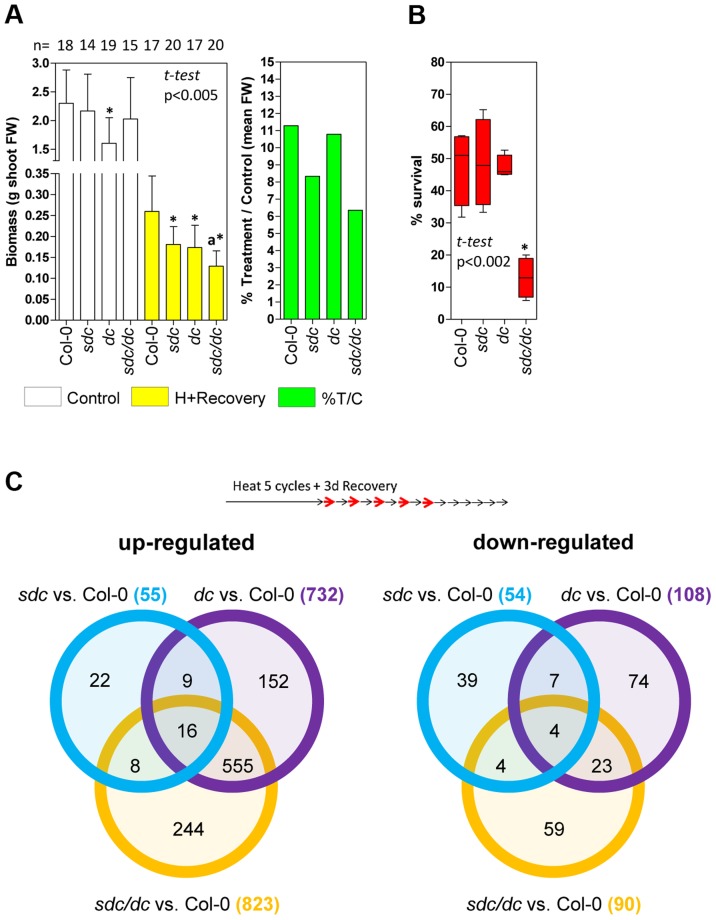
Physiological and transcriptomic data for *sdc*, *dc* and *sdc/dc* mutants. **A**, Left: Physiological tolerance measured as final aerial biomass. Bars represent means ± SD of shoot fresh weigh (FW) of controls and plants treated for 15 heat cycles plus 18 days of recovery under standard conditions. Numbers at the top represent the independent plants tested.^*^ means statistically significant to the corresponding wild type Col-0 control, and ^a^ means statistically significant to the *dc* heat + recovery plants (*P*<0.005, Student *t*-test). Right: Relative mean aerial biomass as percentage of controls obtained from the data shown on the left, denoting a decrease of at least 30% in relative biomass after treatment of genetic backgrounds harboring the *sdc* mutation. **B**, Survival to moderately high temperatures, expressed as percentage of seedling survival after 7 days of continuous heat treatment at 35°C from four independent biologically replicated pools of 30–50 seedlings. Data are depicted as box plots. ^*^ means statistically significant to wild type Col-0 (*P*<0.002, Student *t*-test). **C**, Venn diagrams comparing differentially regulated genomic features in the *sdc*, *dc* and *sdc/dc* mutants. Transcriptome analysis was performed in duplicated biological samples after a 5 heat-cycle entrainment +3 days of recovery (experimental design shown at the top). Venn diagrams show up- or down-regulated genomic features for each mutant compared with the wild-type. Within the diagrams, the numbers represent differentially regulated genomic features either unique or shared, whereas the legend brackets show the total number of up- or down-regulated genomic features.

The *sdc* mutant displays neither seed nor somatic developmental abnormalities [13], [25] and, thus, it's physiological or developmental role remains unknown. However, epigenetic suppression of *SDC* seems to be required for proper plant development. DNA methylation mutants such as *dc* display abnormal phenotypes during vegetative growth, which can be attributed to high ectopic activity of *SDC*, given that the phenotype is suppressed in the *sdc/dc* mutant [13]. Therefore, we tested the effect of *SDC* induction in vegetative tissues under elevated temperatures, comparing the heat stress responses of wild type to the *sdc*, *dc* and *sdc/dc* mutants. Under our standard heat-entrainment/recovery conditions, transcripts levels of typical heat-responsive genes did not change in any of these mutants (Figure S10), indicating unaltered heat perception and signaling. As a consequence, we did not expect any disturbance of heat shock-induced acquired-thermotolerance [27], so we performed a non-lethal long-term heat stress experiment of wild type and *sdc*, *dc* or *sdc/dc*. Seven-day-old seedlings received 15 entrainment heat-cycles followed by 3 days of recovery and were then grown in soil for a further 15 days under standard conditions, before harvesting and determination of their total aerial fresh weight. The *sdc* and *sdc/dc* mutants showed significantly reduced biomass than the corresponding controls, wild-type and *dc* respectively (Figure 5A, left). Absence of a functional *SDC* gene accounted on average for approximately 30% of biomass deficit (Figure 5A, right), suggesting a role for the SDC protein in the response to long-term heat. The growth of *sdc/dc* was more affected by non-lethal heat stress than *sdc* or *dc* separately, suggesting that mutations leading to depletion of CHG and CHH DNA methylation may not behave epistatic to a mutation of *SDC* under specific heat stress treatments. To test this hypothesis in an independent experimental setup, we examined the survival of wild type, *sdc*, *dc*, and *sdc/dc* under moderately high temperatures that resulted in 50% lethality of the wild type [27]. Seedling survival was scored after growth consecutively at 21°C, 35°C, and then 21°C, each for 7 days. The wild-type, *sdc* and *dc* lines showed survival rates of approximately 50% but the survival of *sdc/dc* was significantly lower at 13% (Figure 5B). These results are consistent with at least two parallel pathways contributing to recovery from moderately high temperatures; the first mediated by SDC activity and the second involving epigenetic regulation of CHG/CHH methylation, potentially influencing the transcriptome.

As a consequence, we compared the transcriptomes of wild type, *sdc*, *dc* and *sdc/dc* during recovery from heat stress entrainment (Table S1). Compared with the wild type, a total of 109, 840 and 913 loci were differentially regulated in *sdc*, *dc* and *sdc/dc*, respectively (Figure 5C). Selected genes found to be altered in the transcriptome analysis were validated in an independent experiment, using qRT-PCR (Figure S11). The 109 genes differentially regulated in *sdc* are involved in a wide range of cellular processes (Figure S12A). Of these, 68 were represented on the Affymetrix GeneChip ATH1 microarray used earlier for expression profiling that revealed genes differentially expressed during a prolonged heat treatment that led to release of TGS [14]. Out of these 68 loci, transcript levels of 27 (ca. 40%) changed under long-term constant heat [14] (Table S1), supporting the notion that *SDC* is involved in the regulation of a sub-set of responses to heat stress. Surprisingly, one-third of the genes with altered transcript levels in *sdc* were altered similarly in *dc* (Figure S12B). This raises the possibility that *SDC* controls a sub-set of genes regulated by CHG and CHH methylation. To determine whether this regulation is mediated directly by changes in DNA methylation, we examined available DNA methylation data and observed that none of the loci are subjected to DNA methylation, either in wild-type or in epigenetic mutants [23]. Therefore, the influence on their transcription in both *sdc* and *dc* mutants seems to be indirectly linked to DNA methylation.

Although approximately two-thirds of the loci in *dc* or *sdc/dc* that differed from wild type overlapped and encoded mostly transposons activated in the *dc* mutant, approximately one-third (297) displayed transcriptional changes linked specifically to *sdc/dc* and not shared with either *sdc* or *dc* (Figure 5C). The *sdc/dc* miss-regulated transcripts observed during heat stress recovery are potentially linked to the higher heat sensitivity of the *sdc/dc* mutant. Indeed, 163 of these 297 transcripts were represented on the Affymetrix GeneChip ATH1 microarray used for profiling after long-term heat as described before [14], and 48 of them (ca. 30%) were altered under these condition (Table S1). This is again consistent with the involvement of *SDC* in heat-stress responses, acting together but only partially redundantly with activities involved in the maintenance of CHG and CHH methylation. Taken together, the gene expression profiling data and the observed altered recovery of *sdc* and *sdc/dc* mutants point towards the physiological significance of *SDC* during plant vegetative growth in adverse environmental conditions.

SDC is an F-Box protein putatively involved in ubiquitin-mediated degradation of target proteins by the proteasome [13] but its substrate(s) are unknown. Our attempt to recover potential target interactors using high-throughput tandem-affinity-purification/mass-spectrometry with a TAP-tag fusion [28] with SDC was unsuccessful. However, since the activities of the RdDM pathway and CMT3 that influence *SDC* expression are restricted to the nucleus, we used a vector containing the ubiquitin promoter linked to the coding sequence of SDC fused to the GFP reporter (*UBQ10::SDC-GFP*) in transient transformation assays and obtained clear evidence for the nuclear localization of the SDC-GFP signal (Figure S13).

## Discussion

Epigenetic regulation, typically involving modification of histones and/or remodeling of chromatin, has been implicated previously in plant responses to biotic and abiotic stress [29]. Components of the RdDM pathway and histone deacethylase activity seem to support the survival of plants subjected to lethal heat [30]. Moreover, a heat-sensitive mutant has been isolated in which the heat-induced release of heterochromatic silencing is attenuated [17]. However, the physiological significance of TGS disturbance under long-term heat remained largely unknown, and was suggested previously to be merely a consequence of the thermal disruption of protein-DNA and/or protein-protein interactions [14]. The results presented here suggest that one potential role of silencing release may be the transient expression of epigenetically suppressed loci that encode genes whose activities contribute to stress tolerance. We provide the example of the epigenetically silenced and imprinted gene *SDC*, with a physiological role in responses to long-term heat stress. Our findings suggest that the silencing of *SDC* in vegetative tissues was concealing its involvement in stress responses, when transcriptional reactivation occurs following exposure to heat stress. Interestingly, this appears to take place independently of canonical heat-stress signaling pathways.

The expression of a subset of imprinted loci is restricted to the endosperm and although some imprinted genes are active in seed development and maturation, many of them have as yet no ascribed roles in seeds [11]. The results presented here for *SDC* provide an example that their activity may be revealed under particular growth conditions and that their functions could be executed beyond the tissue of parent-of-origin expression. Furthermore, it may be possible that such concealed activities exist for other imprinted genes, under different stress conditions. For example, it was reported previously that pathogens, UV, cold, and freezing treatments may also transiently disturb epigenetic silencing [15], [31], [32]. In line with this, stress-induced change in DNA methylation has been proposed to impart regulatory control over defense genes that become activated by pathogen attack [32]. Our analyses of published data demonstrated that some endosperm-imprinted genes can be activated by various environmental stresses. Thus, it is plausible that imprinted or epigenetically suppressed loci may exert their activities during vegetative growth upon trigger-specific destabilization of TGS. However, the interactions of particular stress(es)/gene(s) require detailed studies of individual examples.

It was shown previously that heat-induced release of silencing occurs across all plant tissues [16]. However, in the case of *SDC*, TGS destabilization seemed to occur mostly in young, expanding leaves. Together with the decreased shoot biomass observed with the *sdc* mutant following heat stress, these results implicate this gene in the expansion/maturation of leaves of plants exposed to high temperatures. This reinforces the current concept that epigenetic silencing may bring about new and unexpected plasticity to gene regulation and plant phenotypes [33]. As a remarkable example, we also observed that *SDC* activation occurred only above a certain window of absolute temperature, resembling a thermal-sensing mechanism [34]. The epigenetic machinery may, therefore, mediate transcriptional control of certain stress responses in a threshold fashion, as defined previously [35]. However, it is not currently clear whether the *SDC* locus itself senses absolute temperature through its dynamic epigenetic landscape.

We have shown that the kinetics of *SDC* re-silencing following variable repeated heat stress is tailored to the previous entrainment, thus displaying transcriptional memory that is especially apparent in the recovery phase. Such a convoluted transcriptional regulation appears also to rely on post-stress epigenetic resetting, since *SDC* transcripts after heat treatment did not recover to the control levels in epigenetic mutants like *mom1-2*, *ddm1-2* and *met1-1*. This implies that epigenetic regulation in plants stores previous stress experiences during vegetative development. In connection to this, somatic transcriptional memory was demonstrated recently in plants subjected to osmotic stress and this was attributed to dynamic changes in histone modification [36], [37]. Major stress-induced changes in several common histone marks were not observed within the *SDC* locus. Also, since the heat-mediated activation of *SDC* gene occurs in mutants impaired in siRNA biogenesis, a regulatory involvement of siRNAs is unlikely. However, it remains possible that other not tested histone modifications or physical properties of chromatin are responsible for the memory phenomenon. Previously, it have been demonstrated that the transient release of epigenetic suppression under heat stress coincides with decreased nucleosome occupancy, and that chromatin remodelling or assembly factors are a requirement for the fast restoration of silencing [14], [38]. Although these mechanisms may contribute to the regulation of *SDC*, publically available data suggest that nucleosome density in its promoter area is already low without heat stress [39].

The SDC protein is present in cell nuclei and belongs to the F-Box protein family, which mediates ubiquitin-tagged degradation of proteins and are among the fastest evolving gene families in plants [40]. It is therefore possible that SDC targets a yet unknown nuclear protein for degradation. The *A. thaliana* gene *Upward Curly Leaf 1* (*UCL1*), which encodes a protein very similar to SDC, has been shown to target *Curly Leave* (CLF) [41]. CLF is a histone-methyl-transferase of the polycomb-repressive-complex-2 (PRC2), which is involved in various aspects of sporophyte development [42]. Despite an intensive search, we failed to reveal a SDC substrate but CLF or CLF-related proteins remain as potential candidates. Regardless of the actual target protein(s), our transcriptome analysis pointed towards the involvement of SDC in the transcriptional regulation of a sub-set of genes responding to long-term heat.

**Figure 6:**
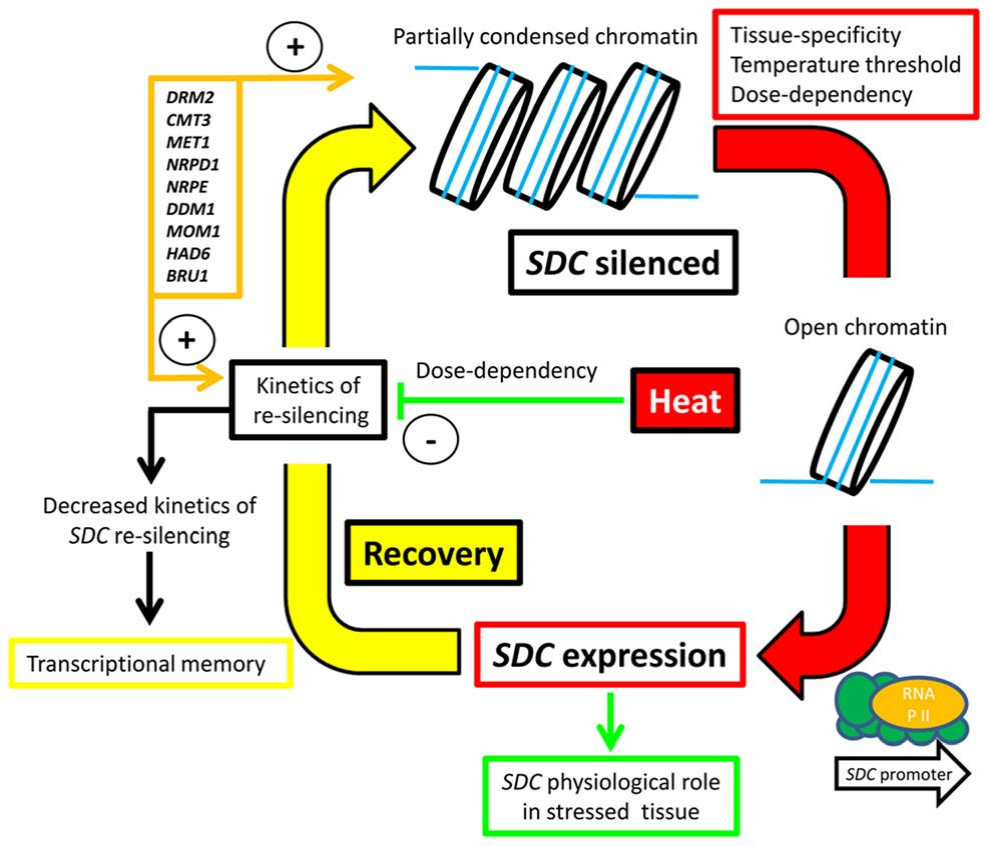
Hypothesized silence/de-silence loop model showing the different steps of transcriptional epigenetic control in the heat-induced expression of the *SDC* gene.

We propose a silencing/de-silencing loop model illustrating the thermal control of *SDC* expression (Figure 6). In this model, heat-induced destabilization of the suppressive chromatin allows the transcriptional machinery to access the *SDC* promoter, but this occurs only above a particular window of absolute temperature. Moreover, the level of transcriptional activation depends on the severity and duration of the heat stress. Expression of *SDC* leading to synthesis and nuclear translocation of the SDC protein subjects certain nuclear protein(s) to ubiquitin-mediated degradation. Following termination of the heat stress, slow *SDC* re-silencing allows a temporal extension of SDC activity. All these regulatory mechanisms occur independently and in parallel to canonical heat-shock perception and signaling, but rely on epigenetic properties. It is likely that these arise through the targeting of TGS to the tandem-repeats residing in the *SDC* promoter. We propose that two steps, the emergence of these repeats and the subsequent epigenetic control, led to rapid evolution of a novel type of environmentally regulated transcriptional output.

## Materials and Methods

### Plant material, growth and experimental conditions

All *Arabidopsis thaliana* Col-0 mutants used in this study have been described and characterized previously: *sdc*, *dc* (*drm2-2/cmt3-11*) and *sdc/dc* [13], *met1-1* [43], *mom1-2* [18], *ddm1-2* [1], *nrpd1-3* and *nrpd2a-2/2b-1* [44], *nrpe1-2* [45], *kyp-7* [4], *suvh2* [46], *bru1-4* [23], *rts1-1* (*HAD6*, Aufsatz et al. [47]), and *hot1-3* [21]. As control wild type, we used the Col-0 line N22681 (The Nottingham Arabidopsis Stock Centre, NASC). The *L5-GUS* silenced transgenic is the b5b line of Morel et al. [2], and the LUC+ positive control was the *UBQ3::LUC+* line (named LUC26) from Yokthongwattana et al. [20]. The different *A.thaliana* accessions are available at ABRC (http://abrc.osu.edu/) and NASC (http://arabidopsis.info/).

Seeds were surfaced sterilized and sown in sealed petri dishes with 0.5× MS medium containing 1% sucrose, 0.8% agar, and 0.05% MES at pH 5.7. Stratification was applied for 3 days at 4°C. Seedlings were grown for 7 or 12 days at 21°C in a CU-22L growth chamber (Percival) with a 12 h/12 h (day/night) light cycle (termed “standard conditions”), and then subjected to changes in temperature only during the light period (with the exception of the heat time-course and the survival under moderately high temperatures experiments). The experimental design for each particular experiment is shown at the top of the corresponding graphs in the Results section. In all cases, the light cycle was maintained. Heat always means 37°C unless otherwise stated. Long-term heat-entrainment experiments consisted of a varying number of heat cycles (day at 37°C, night at 21°C, 12/12 h), followed by 3 days of recovery at 21°C and a second stress treatment of 37°C for 12 h. For the non-lethal long-term heat experiment, seedlings were subjected to 15 days of heat cycles followed by 3 days of recovery. Plants were then transplanted to soil for a further 15 days of recovery in a growth room at 21°C, after which total above-ground fresh weight was determined. For survival under moderately high temperatures, seedlings were growth *in vitro* consecutively at 21°C, 35°C, and then 21°C, each for 7 days [27].

### Gene expression analysis

Total RNA was isolated using the RNeasy Plant Mini kit (Invitrogen). cDNA synthesis and quantitative real-time RT-PCR analysis were performed as described previously [48] using the geometric mean of three housekeeping genes for normalization. In short, 5 ug of total RNA was treated with TURBO DNA-free kit (Ambion) and first-strand cDNA was synthesized with an oligo dT primer using the SuperScript III First-Strand Synthesis System (Invitrogen). Real-time PCR was performed with the Power SYBR Green PCR Master Mix (Applied Biosystems) in a final reaction volume of 10 µl and with a 1/10 dilution of the cDNA. Cycling and dissociation curves were analyzed in an ABI PRISM 7900HT Sequence Detection System (Applied Biosystems). Primer design, reaction parameters and analysis of expression data were performed as described previously [49], [50]. We used the geometric mean of three housekeeping genes for normalization; these were *UBQ10* (*AT4G05320*), *SAND* (*AT2G28390*) and *PDF2* (*AT1G13320*). However, only *SAND* and *PDF2* were used as housekeeping genes for comparisons across *A. thaliana* accessions. Typical heat-responsive genes were selected from those showing high transcriptional induction under long-term heat [14]. A list of the primers used is available in Table S2.

Whole transcriptome analysis was performed by the Functional Genomics Center of ETH University (Zurich, Switzerland) using a HiSeq 2000/2500 (Illumina) platform to perform unstranded RNA-seq from purified poly-A RNA obtained from duplicated biological replicates. For annotation, mapping of reads was carried out using gene models from the TAIR10 genome assembly (http://www.arabidopsis.org/). Statistical analysis was performed with the edgeR Bioconductor package and the false-discovery-rate (FDR) was computed with the Benjamini-Hochberg algorithm. A genomic feature was considered differentially changed in a mutant versus wild type comparison when the Benjamini-Hochberg's FDR was <0.1 and the log2 fold change was >1 or <−1. Raw GeneChip Arabidopsis ATH1 Genome Array (Affymetrix) data were analyzed with the RobiNA software [51] and the probesets were considered differentially changed by the heat treatment using the above parameters. The non-redundant functional categories of the differentially changed features were assessed with the MapMan software [52]. Mean-normalized expression data of confirmed endosperm-imprinted genes in seedlings under stress was taken from Kilian et al. [26].

### Vector design and transgenic plants

For cloning purposes, PCR was performed using the Phusion High-Fidelity DNA Polymerase (NEB) and blunt-end products were cloned using the CloneJET PCR Cloning Kit (Thermo Scientific). A fragment of the *SDC* promoter (1198 bp upstream of the ORF) was PCR amplified from genomic DNA, cloned, sequenced, and re-cloned into a *pGPTVII-bar-MCS* (multi-cloning-site) plasmid using the BamHI/XhoI sites [53], producing the *pGPTVII-bar-1200PromSDC* vector. *LUC+* was PCR amplified from genomic DNA of a *UBQ3::LUC+* line [20], cloned, sequenced, and re-cloned into the previous construct using the XhoI/XmaI sites, thus producing the *-1200PromSDC::LUC+* construct. The ORF of *SDC* without a stop codon was PCR amplified from genomic DNA, cloned, sequenced, and re-cloned into the *pGPTVII-bar-UBQ10-GFP5* using the BamHI/XhoI sites in frame with the GFP5, giving the *UBQ10::SDC-GFP* construct. All original pGPTVII binary plasmids were kindly provided by Dr. Rainer Waadt (University of California SD, USA). A list of the primers used is available in Table ST2. The *Agrobacterium tumefaciens* pGV3101 strain was used to transform *A. thaliana* using the standard floral-dip method. Transgenic lines were selected *in vitro* for resistance to BASTA (dl-phosphinothricin, Duchefa).

### Other methods

*In vivo* measurements of luciferase activity were performed by spraying the treated transgenic seedlings with luciferin (Biosynth, 1 mM in water). After 5 min in the dark, images were captured with a CDD ORCA2 C4742-98 digital camera (Hamamatsu) and then analyzed with Wasabi Imaging software. Luciferase activity was detected without a filter, whereas a 632.8 nm filter and blue light was used to detect the chlorophyll signal.

DNA methylation was analyzed by cloning and sequencing of PCR products from bisulfite-treated genomic DNA, from whole young seedlings subjected to entrainment by 5 heat cycles along the corresponding controls. Genomic DNA was isolated by standard CTAB buffer and further fenol-chloroform extractions and precipitation. Bisulfite treatment was performed with the Epitect Bisulfite Kit (Quiagen). PCR products were amplified with Taq polymerase (Promega) using a touch-down PCR strategy and cloned with the pGEM-T Easy Vector System I (Promega). Primer design and analysis of sequences with differentially methylated cytosines were performed with Kismeth and CyMATE [54], [55]. Samples used to assess histone modifications in the *SDC* locus were kindly provided by Dr. Herve Gaubert (University of Cambridge, UK). Chromatin immunoprecipitation was performed in tissue from whole young seedlings following a protocol adapted from Gendrel et al. [56] and Nelson et al. [57]. A list of the primers used to test these samples is available in Table ST2.

For cellular localization of the SDC-GFP fusion protein, 4-week-old *Nicotiana benthamiana* plants were infiltrated with *A. tumefaciens* carrying the corresponding constructs according to Schütze et al. [58]. After 3 days, pieces of leaves were mounted and the GFP signal from transiently transformed epidermal cells photographed with a confocal LSM 700 laser scanning microscope (Zeiss) housed by the UNIGE Bioimaging Core Facilities (http://www.unige.ch/medecine/bioimaging/index.html).

## Supporting Information

Figure S1 Transcript levels of typical heat-responsive genes (HSP20, HSP101, APX2 and HSP70) and partially-silenced genes (SQN and APUM9) in transcriptional memory experiments (Experimental design showed at the top-left corner). In all cases, note that there is no increase in the recovery transcript level correlating with the number of heat-cycles. Bars represent means ± SE as a log2 ratio with the non-treated wild-type Col-0 control conditions (i.e., Col-0 control  = 0); replicated samples were pooled from 40–60 whole seedlings.
(PDF)

Figure S2 **A**, Proposed model to explain the correlation between the recovery transcript level and the number of heat-cycles in the entrained L5-GUS and SDC, based on a change in the kinetics of re-silencing. **B**, Kinetics of transcript level re-silencing of exemplified transposable elements in the recovery phase following a 5 heat-cycle entrainment. Dots represent mean gene expression as a log2 ratio with the non-treated control condition (i.e., control  = 0); replicated samples were pooled from 40–60 whole seedlings.
(PDF)

Figure S3 **A**, Transcript levels of typical heat-responsive genes (*HSP20*, *HSP101* and *HSP70*) in different tissue of transgenic Col-0 seedlings (*-1200SDCProm::LUC+*) after 5 heat-cycles entrainment. Bars represent means ± SE as a log2 ratio with the non-treated wild-type Col-0 control conditions (i.e., Col-0 control  = 0); replicated samples were pooled from 20–30 seedlings (E =  epicotyl, H =  hypocotyl). **B**, Transcript levels of *LUC+* in transgenic Col-0 seedlings (*-1200SDCProm::LUC+*) after 5 heat-cycles entrainment and recovery (design showed at the top). Note the similarities between *LUC+* and *SDC* transcriptional patterns. Dots represent mean as a log2 ratio with the non-treated wild-type Col-0 control conditions (i.e., Col-0 control  = 0); replicated samples were pooled from 40–60 whole seedlings. **C**, *In vivo* luciferase activity in 15-days old Col-0 transgenic seedlings (-*1200SDCProm::LUC+*) after an entrainment of 5 heat-cycles and varying recovery times. LUC: luciferase signal, CHL: chlorophyll signal. The age of plants and treatments are shown at the top. LUC+  =  luciferase signal, CHL  =  chlorophyll signal.
(PDF)

Figure S4 Transcript levels of *SDC* across selected *A.thaliana* accesions, under non-treated control conditions, after a 5 heat-cycle entrainment, and after 5 heat-cycle entrainment +3 days recovery. Bars represent means ± SE as a log2 ratio with the non-treated wild-type Col-0 control conditions (i.e., Col-0 control  = 0); replicated samples were pooled from 40–60 whole young seedlings.
(PDF)

Figure S5 **A** and **B**, Test for thermal threshold in the activation of *LUC+* in transgenic Col-0 (*-1200SDCProm::LUC+*) and typical heat-responsive genes (*HSP101, HSP70 and APX2*), in *A.thaliana* seedlings subjected to different experimental designs depicted at the top of each graph. **A**, Seedlings were grown at standard conditions (21C day/nigh 12/12 hs) for 7 days and then subjected to daily increased growing temperature. **B**, Seedlings were grown at normal condition for 12 days, but then subjected to one-step increase in the growing temperature for 12 h. Bars represent the mean ± SE gene expression as a log2 ratio to the non-treated wild-type Col-0 control condition (i.e. Col-0 control  = 0), from replicated samples pooled from 40–60 whole seedlings. **C** and **D**, Transcript levels of *LUC+* in the transgenic Col-0 (*-1200SDCProm::LUC+*) and typical heat-responsive genes (*HSP101, HSP70 and APX2*) of *A.thaliana* seedlings subjected to different heat-time course experiments. **C**, Seedlings were subjected to constant heat at 37C, and harvested at different time points. **D**, Seedlings were grown at normal condition for 12 days, and then subjected in one-step to constant 32C or 34C for long periods of time. Dots represent the mean as log2 ratio with the non-treated control conditions (i.e., control  = 0); replicated samples were pooled from 40–60 seedlings. The experimental designs are shown at the top of each graph. Note the similarities between *LUC+* and *SDC* transcript levels in each corresponding experiment shown also in Figure 3.
(PDF)

Figure S6 Transcript levels of typical heat-responsive genes (*HSP20, HSP101* and *APX2*) across *A.thaliana* epigenetic mutants under non-treated control conditions, 5 heat-cycles entrainment and 5 heat-cycles entrainment +3 days recovery. Bars represent the means ± SE as a log2 ratio with the non-treated control conditions (i.e., control  = 0); replicated samples were pooled from 40–60 seedlings.
(PDF)

Figure S7 DNA methylation pattern of the *SDC* locus in samples of young whole seedlings subjected to control conditions and 5 heat-cycle entrainment. PCR products used to reveal different portions spanning the *SDC* locus are shown at the top. In each case, 10 clones of bisulfite-treated genomic DNA were sequenced.
(PDF)

Figure S8 Chromatin IP of different histone-modification in *A.thaliana* young whole seedlings subjected to control conditions or heat stress. PCR products used to reveal different portions spanning the *SDC* locus are shown at the top. Input signals for each IP are also displayed.
(PDF)

Figure S9 Differential expression of 93 endosperm-imprinted genes under different stress situations. Data taken from Hiseh *et al*. (2011) and Killian *et al*. (2007).
(PDF)

Figure S10 Transcript levels of typical heat-responsive genes (*HSP20, HSP101* and *APX2*) across *A.thaliana* wild-type Col-0 and *sdc*, *dc* and *sdc/dc* mutants under non-treated control conditions, after a 5 heat-cycle entrainment, and a 5 heat-cycle entrainment +3 days recovery. Bars represent means ± SE as a log2 ratio with the non-treated wild-type Col-0 control conditions (i.e., Col-0 control  = 0); replicated samples were pooled from 40–60 whole seedlings.
(PDF)

Figure S11 Validation of transcriptomic results. RNA-seq data is expressed as normalized read counts (RC) of duplicated biological replicates (mean±SD). An independent experiment was used for qRT-PCR analysis (control, 5 heat-cycles entrainment and various recovery times, experimental design shown at the top). In this case, bars represent means ± SE as a log2 ratio with the non-treated wild-type Col-0 control conditions (i.e., Col-0 control  = 0); replicated samples were pooled from 40–60 whole seedlings.
(PDF)

Figure S12 Transcriptomic results. **A**, Overview of functional categories of genes changed in the *sdc* mutant during the recovery from heat. Transcriptome analysis was performed in biologically duplicated samples after 5 heat-cycles entrainment +3 days of recovery (experimental design shown at the top). 109 genomic features were considered significantly changed in *sdc* vs. wild-type (with a FDR<0.1 and a Log2 fold change >1 or <−1), and were sorted in non-redundant functional categories using the MapMan software. **B**, Correlation of relative transcript levels between the differentially changed genomic features shared by *sdc* and *dc* mutants.
(PDF)

Figure S13 Transiently transformed *N. benthamiana* epidermal cells with a positive control (*UBQ10::GFP*, left) and a SDC-GFP protein fusion (*UBQ10::SDC-GFP*, right). Note the clear nuclear localization of the SDC-GFP fusion protein.
(PDF)

Table S1 Transcriptomic results. RNA-seq data is expressed as normalized read counts (RC) of duplicated biological replicates (mean±SD). The differentially expressed genes during a prolonged heat treatment, leading to release of TGS, were taken from Pecinka et al. (2010).
(XLSX)

Table S2 List of the primers used.
(XLSX)
