# Supplementary figures and images for: Heat-Induced Release of Epigenetic Silencing Reveals the Concealed Role of an Imprinted Plant Gene
Source: PLoS Genet. 2014 Nov 20;10(11):e1004806. doi: 10.1371/journal.pgen.1004806 (PMC4238952; doi:10.1371/journal.pgen.1004806)

Figure S1

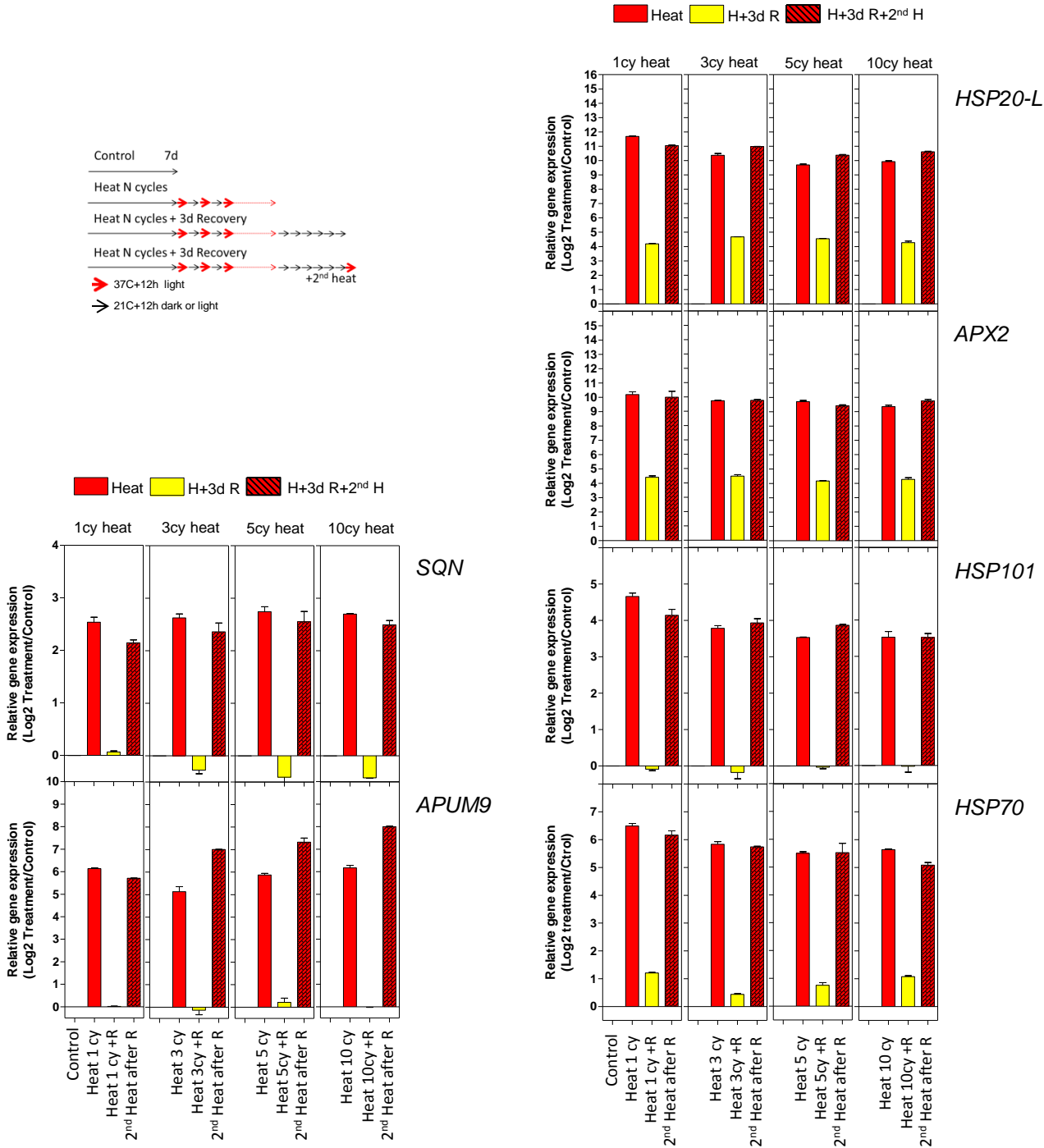

Supplement: Figure S1 — Transcript levels of typical heat-responsive genes (HSP20, HSP101, APX2 and HSP70) and partially-silenced genes (SQN and APUM9) in transcriptional memory experiments (Experimental design showed at the top-left corner). In all cases, note that there is no increase in the recovery transcript level correlating with the number of heat-cycles. Bars represent means ± SE as a log2 ratio with the non-treated wild-type Col-0 control conditions (i.e., Col-0 control = 0); replicated samples were pooled from 40–60 whole seedlings. (PDF) [file pgen.1004806.s001.pdf]

Figure S2

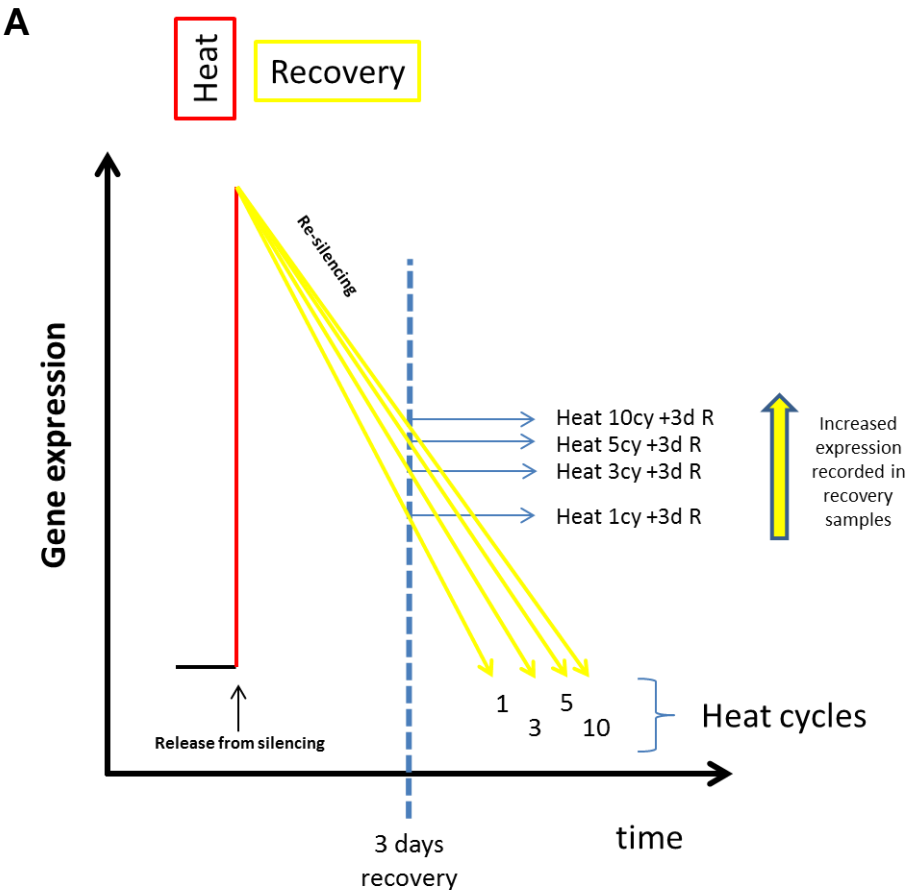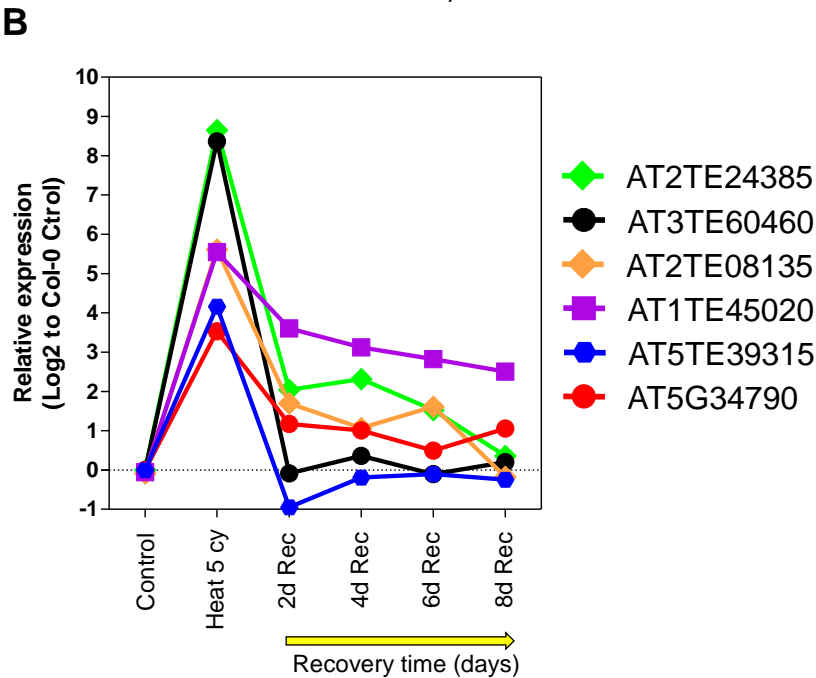

Supplement: Figure S2 — A, Proposed model to explain the correlation between the recovery transcript level and the number of heat-cycles in the entrained L5-GUS and SDC, based on a change in the kinetics of re-silencing. B, Kinetics of transcript level re-silencing of exemplified transposable elements in the recovery phase following a 5 heat-cycle entrainment. Dots represent mean gene expression as a log2 ratio with the non-treated control condition (i.e., control = 0); replicated samples were pooled from 40–60 whole seedlings. (PDF) [file pgen.1004806.s002.pdf]

Figure S3

A

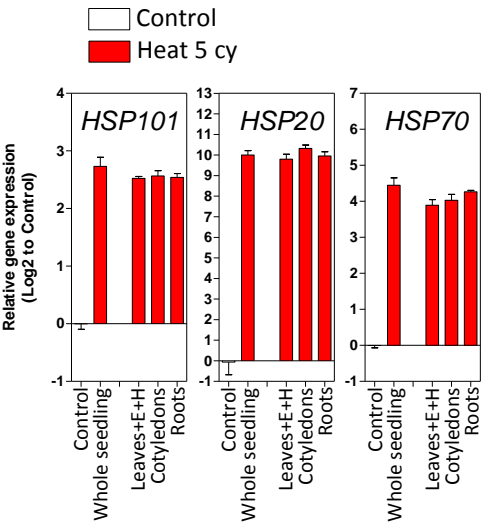

B

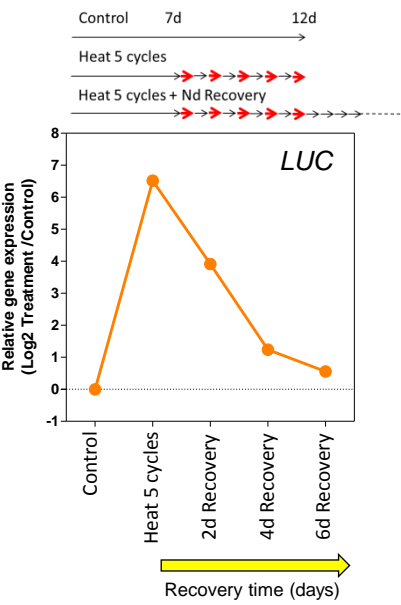

C

5 cycles heat 20 days old +2d recovery 22 days old +4d recovery 24 days old +6d recovery 26 days old

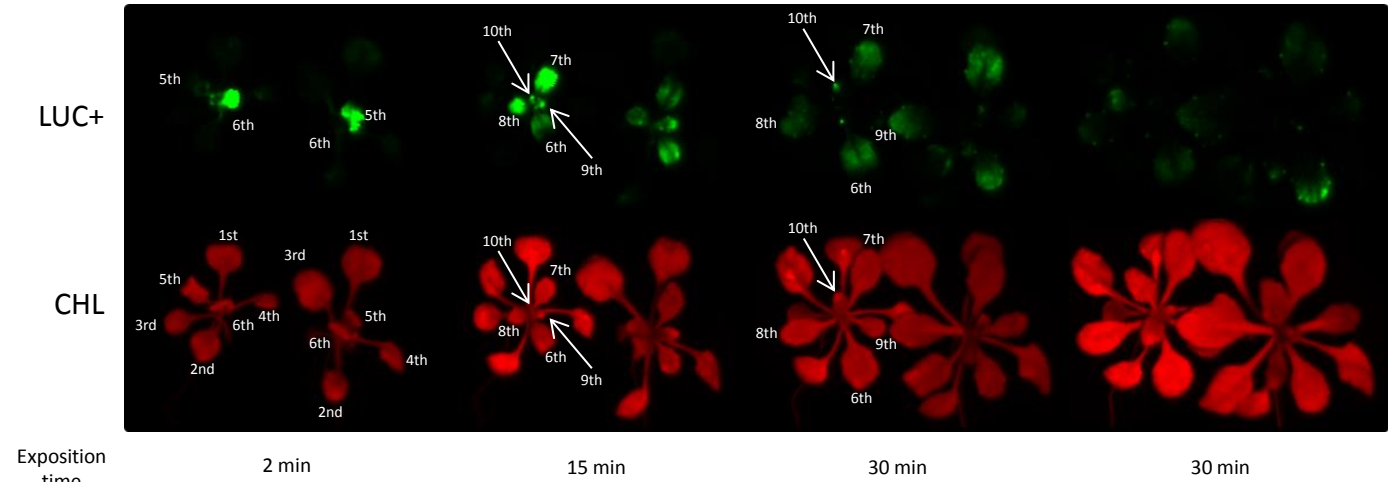

Supplement: Figure S3 — A, Transcript levels of typical heat-responsive genes (HSP20, HSP101 and HSP70) in different tissue of transgenic Col-0 seedlings (-1200SDCProm::LUC+) after 5 heat-cycles entrainment. Bars represent means ± SE as a log2 ratio with the non-treated wild-type Col-0 control conditions (i.e., Col-0 control = 0); replicated samples were pooled from 20–30 seedlings (E = epicotyl, H = hypocotyl). B, Transcript levels of LUC+ in transgenic Col-0 seedlings (-1200SDCProm::LUC+) after 5 heat-cycles entrainment and recovery (design showed at the top). Note the similarities between LUC+ and SDC transcriptional patterns. Dots represent mean as a log2 ratio with the non-treated wild-type Col-0 control conditions (i.e., Col-0 control = 0); replicated samples were pooled from 40–60 whole seedlings. C, In vivo luciferase activity in 15-days old Col-0 transgenic seedlings (-1200SDCProm::LUC+) after an entrainment of 5 heat-cycles and varying recovery times. LUC: luciferase signal, CHL: chlorophyll signal. The age of plants and treatments are shown at the top. LUC+ = luciferase signal, CHL = chlorophyll signal. (PDF) [file pgen.1004806.s003.pdf]

Figure S4

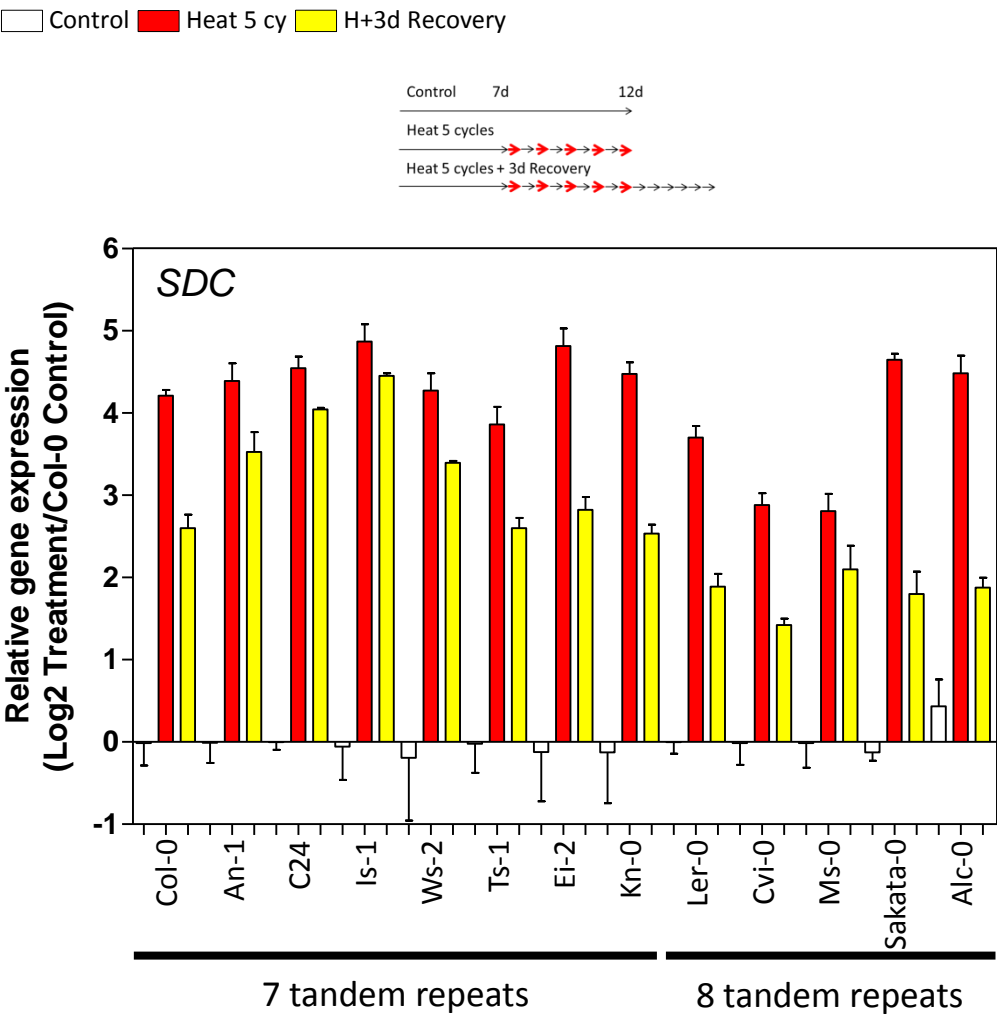

Supplement: Figure S4 — Transcript levels of SDC across selected A.thaliana accesions, under non-treated control conditions, after a 5 heat-cycle entrainment, and after 5 heat-cycle entrainment +3 days recovery. Bars represent means ± SE as a log2 ratio with the non-treated wild-type Col-0 control conditions (i.e., Col-0 control = 0); replicated samples were pooled from 40–60 whole young seedlings. (PDF) [file pgen.1004806.s004.pdf]

Figure S5

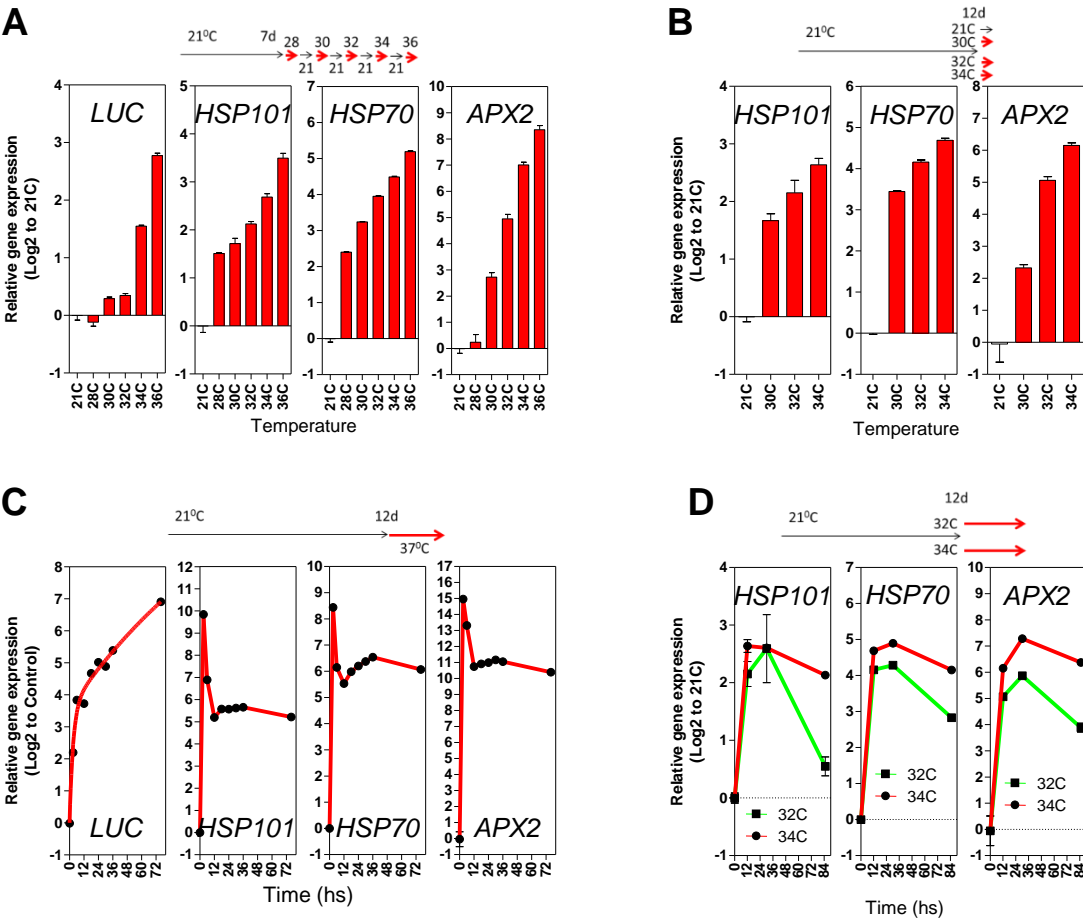

Supplement: Figure S5 — A and B, Test for thermal threshold in the activation of LUC+ in transgenic Col-0 (-1200SDCProm::LUC+) and typical heat-responsive genes (HSP101, HSP70 and APX2), in A.thaliana seedlings subjected to different experimental designs depicted at the top of each graph. A, Seedlings were grown at standard conditions (21C day/nigh 12/12 hs) for 7 days and then subjected to daily increased growing temperature. B, Seedlings were grown at normal condition for 12 days, but then subjected to one-step increase in the growing temperature for 12 h. Bars represent the mean ± SE gene expression as a log2 ratio to the non-treated wild-type Col-0 control condition (i.e. Col-0 control = 0), from replicated samples pooled from 40–60 whole seedlings. C and D, Transcript levels of LUC+ in the transgenic Col-0 (-1200SDCProm::LUC+) and typical heat-responsive genes (HSP101, HSP70 and APX2) of A.thaliana seedlings subjected to different heat-time course experiments. C, Seedlings were subjected to constant heat at 37C, and harvested at different time points. D, Seedlings were grown at normal condition for 12 days, and then subjected in one-step to constant 32C or 34C for long periods of time. Dots represent the mean as log2 ratio with the non-treated control conditions (i.e., control = 0); replicated samples were pooled from 40–60 seedlings. The experimental designs are shown at the top of each graph. Note the similarities between LUC+ and SDC transcript levels in each corresponding experiment shown also in Figure 3. (PDF) [file pgen.1004806.s005.pdf]

Figure S6

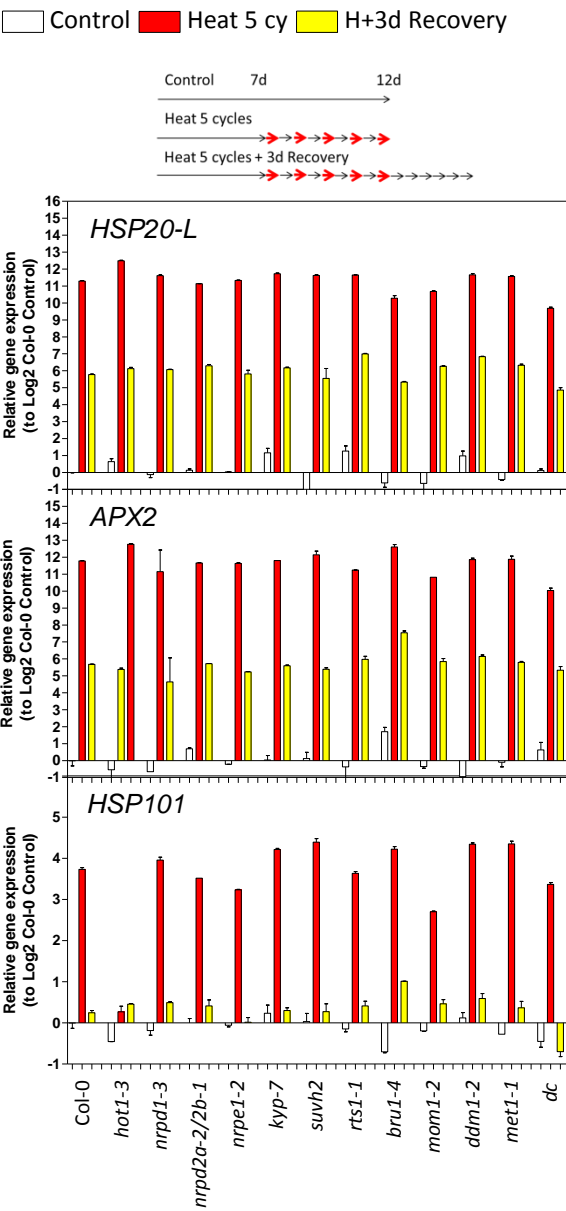

Supplement: Figure S6 — Transcript levels of typical heat-responsive genes (HSP20, HSP101 and APX2) across A.thaliana epigenetic mutants under non-treated control conditions, 5 heat-cycles entrainment and 5 heat-cycles entrainment +3 days recovery. Bars represent the means ± SE as a log2 ratio with the non-treated control conditions (i.e., control = 0); replicated samples were pooled from 40–60 seedlings. (PDF) [file pgen.1004806.s006.pdf]

Figure S7

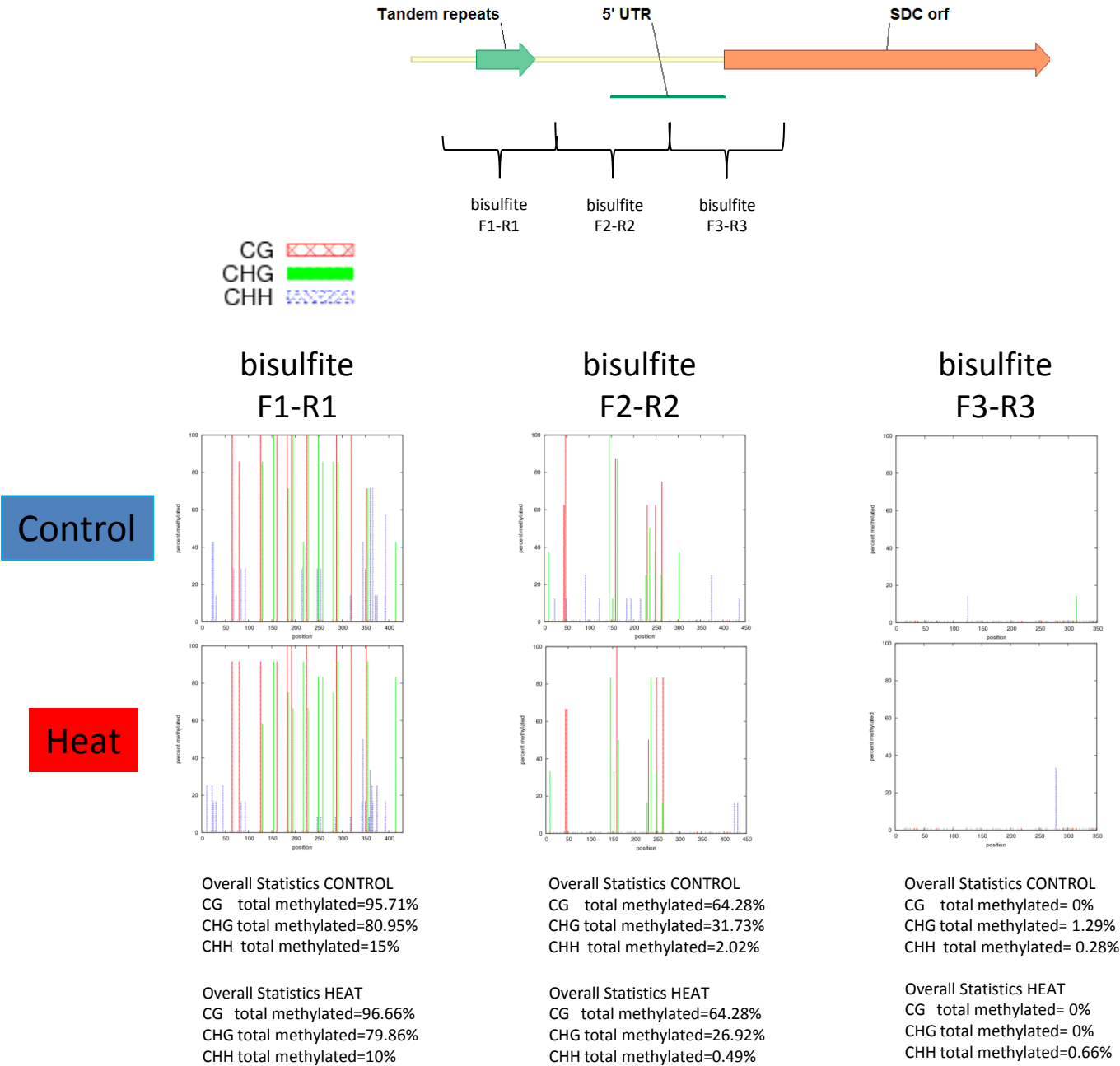

Supplement: Figure S7 — DNA methylation pattern of the SDC locus in samples of young whole seedlings subjected to control conditions and 5 heat-cycle entrainment. PCR products used to reveal different portions spanning the SDC locus are shown at the top. In each case, 10 clones of bisulfite-treated genomic DNA were sequenced. (PDF) [file pgen.1004806.s007.pdf]

Figure S8

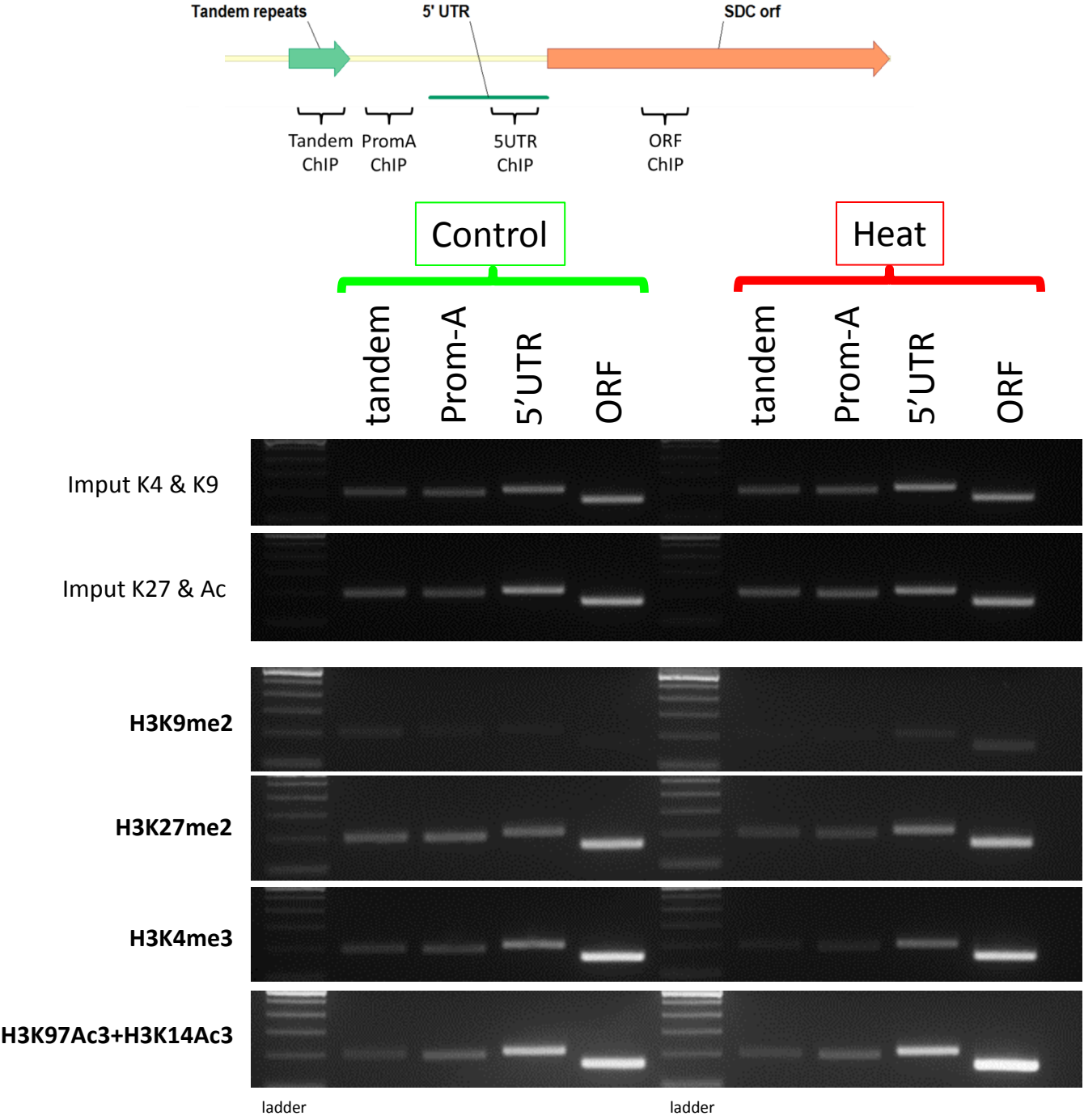

Supplement: Figure S8 — Chromatin IP of different histone-modification in A.thaliana young whole seedlings subjected to control conditions or heat stress. PCR products used to reveal different portions spanning the SDC locus are shown at the top. Input signals for each IP are also displayed. (PDF) [file pgen.1004806.s008.pdf]

Figure S9

Relative gene expression  
(Log2 Treatment/Control average mean-normalized)

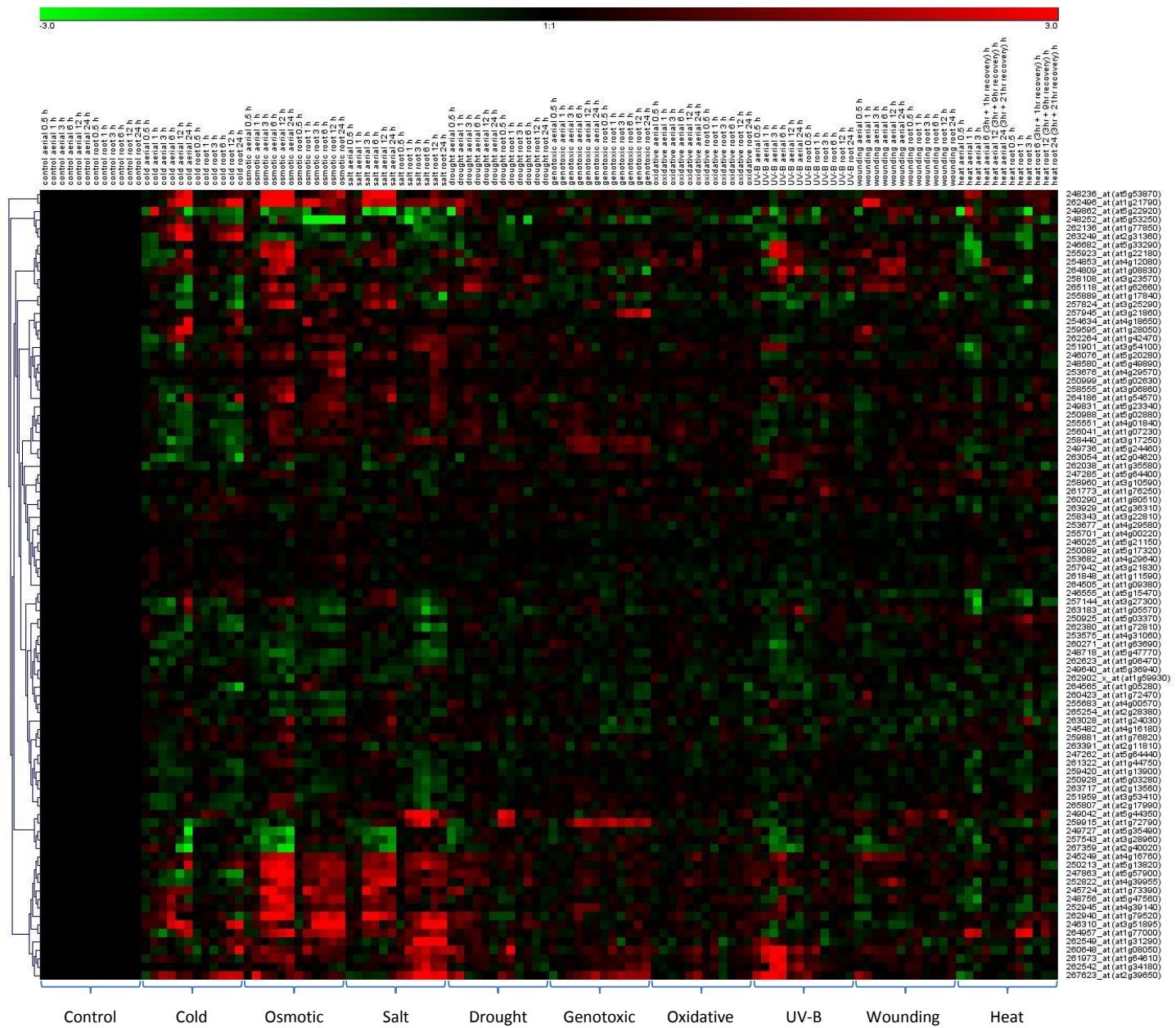

Supplement: Figure S9 — Differential expression of 93 endosperm-imprinted genes under different stress situations. Data taken from Hiseh et al. (2011) and Killian et al. (2007). (PDF) [file pgen.1004806.s009.pdf]

Figure S10

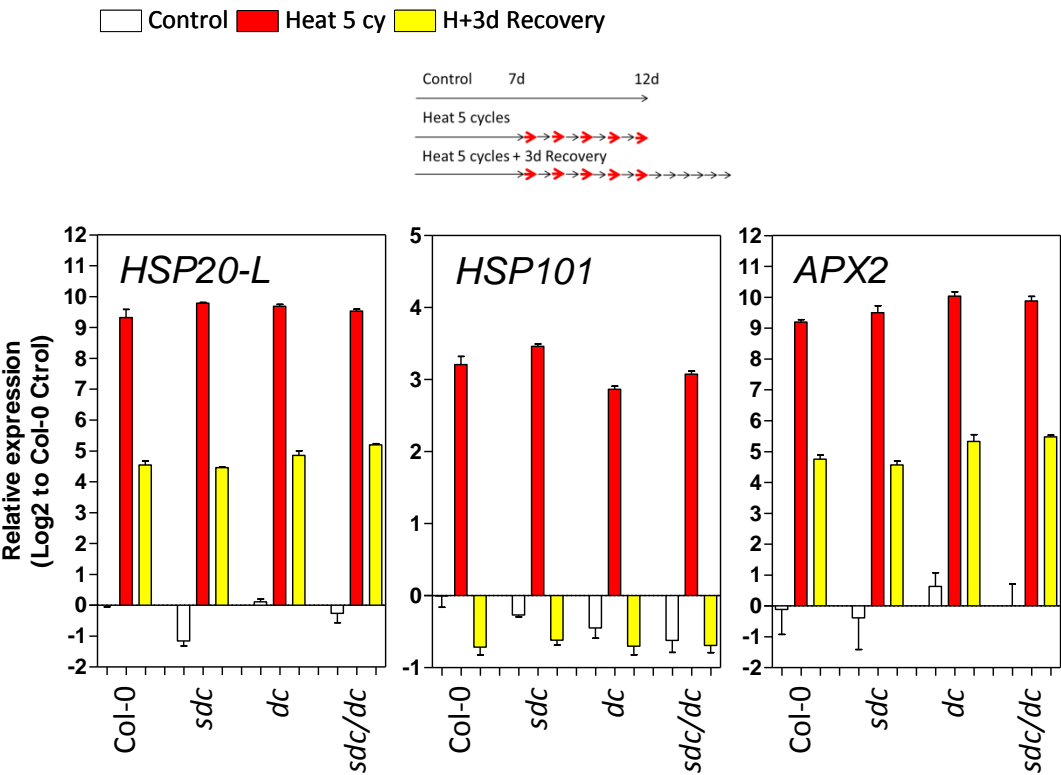

Supplement: Figure S10 — Transcript levels of typical heat-responsive genes (HSP20, HSP101 and APX2) across A.thaliana wild-type Col-0 and sdc, dc and sdc/dc mutants under non-treated control conditions, after a 5 heat-cycle entrainment, and a 5 heat-cycle entrainment +3 days recovery. Bars represent means ± SE as a log2 ratio with the non-treated wild-type Col-0 control conditions (i.e., Col-0 control = 0); replicated samples were pooled from 40–60 whole seedlings. (PDF) [file pgen.1004806.s010.pdf]

Figure S11

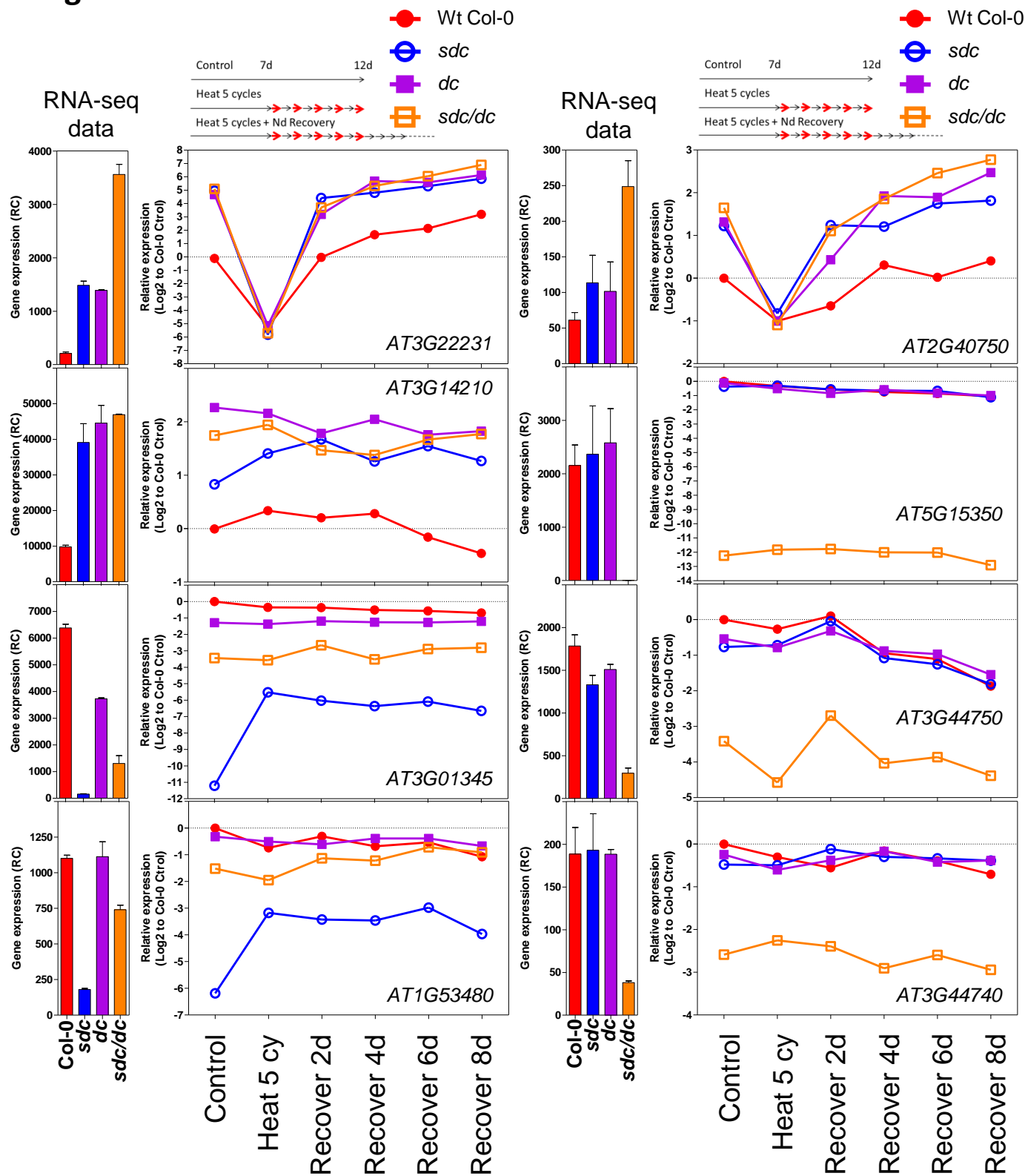

Supplement: Figure S11 — Validation of transcriptomic results. RNA-seq data is expressed as normalized read counts (RC) of duplicated biological replicates (mean±SD). An independent experiment was used for qRT-PCR analysis (control, 5 heat-cycles entrainment and various recovery times, experimental design shown at the top). In this case, bars represent means ± SE as a log2 ratio with the non-treated wild-type Col-0 control conditions (i.e., Col-0 control = 0); replicated samples were pooled from 40–60 whole seedlings. (PDF) [file pgen.1004806.s011.pdf]

Figure S12

A

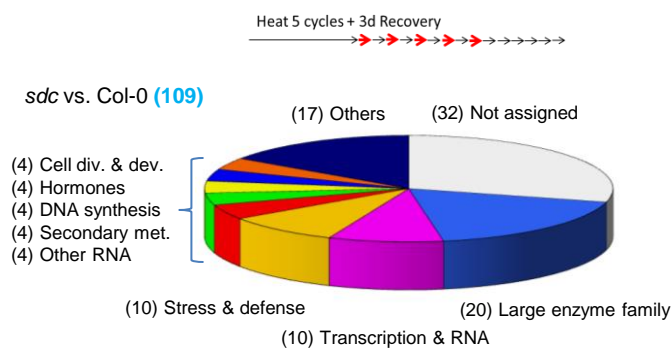

B

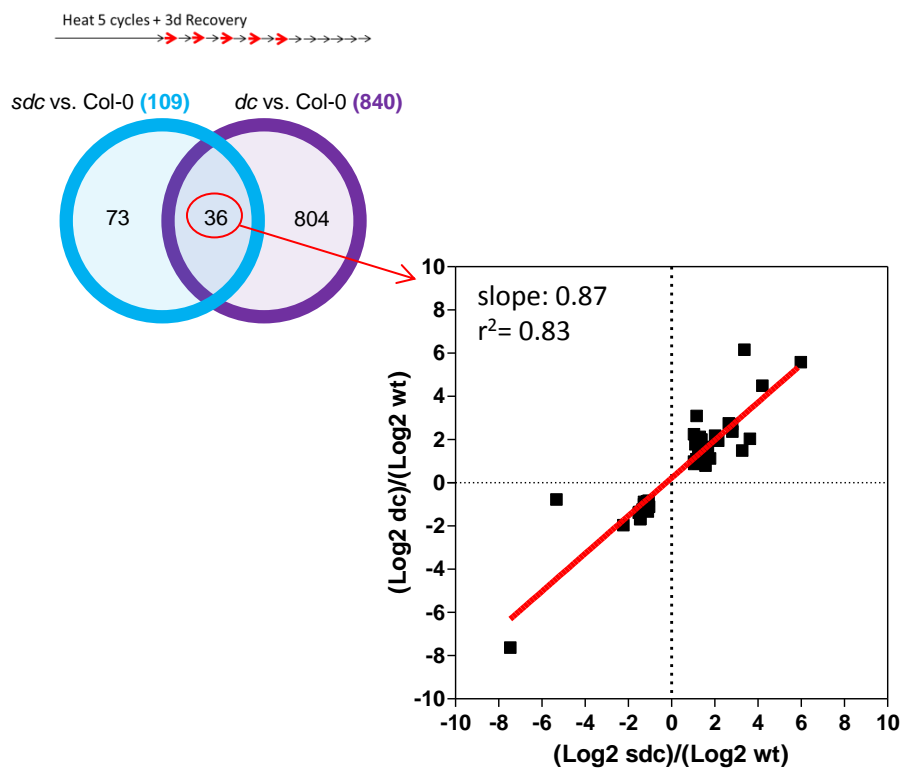

Supplement: Figure S12 — Transcriptomic results. A, Overview of functional categories of genes changed in the sdc mutant during the recovery from heat. Transcriptome analysis was performed in biologically duplicated samples after 5 heat-cycles entrainment +3 days of recovery (experimental design shown at the top). 109 genomic features were considered significantly changed in sdc vs. wild-type (with a FDR<0.1 and a Log2 fold change >1 or <−1), and were sorted in non-redundant functional categories using the MapMan software. B, Correlation of relative transcript levels between the differentially changed genomic features shared by sdc and dc mutants. (PDF) [file pgen.1004806.s012.pdf]

Figure S13

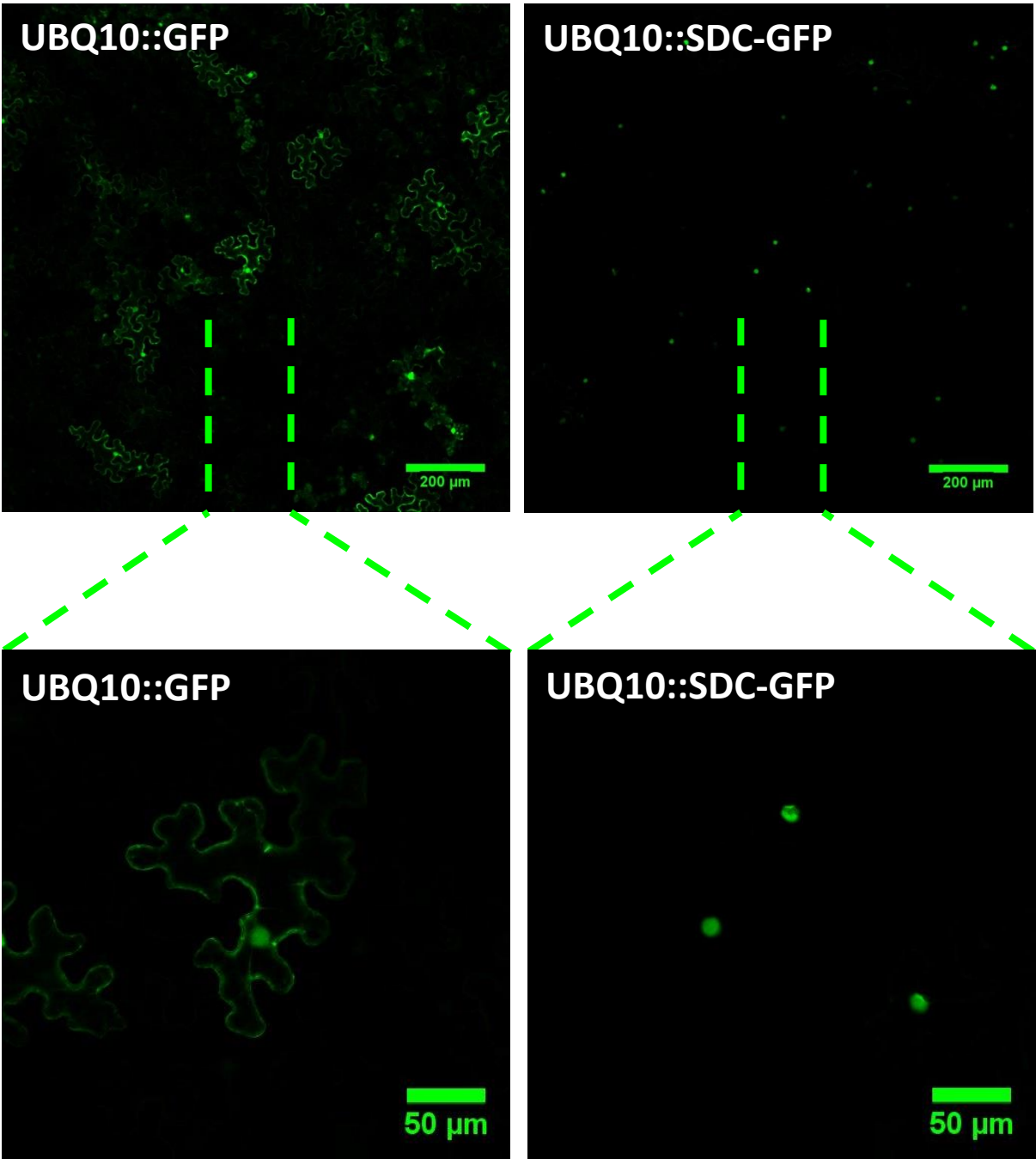

Supplement: Figure S13 — Transiently transformed N. benthamiana epidermal cells with a positive control (UBQ10::GFP, left) and a SDC-GFP protein fusion (UBQ10::SDC-GFP, right). Note the clear nuclear localization of the SDC-GFP fusion protein. (PDF) [file pgen.1004806.s013.pdf]
